# Evaluating the adverse outcome of subtypes of heart failure with preserved ejection fraction defined by machine learning: A systematic review focused on defining high risk phenogroups

**DOI:** 10.17179/excli2021-4572

**Published:** 2022-02-22

**Authors:** Simon W. Rabkin

**Affiliations:** 1 University of British Columbia

**Keywords:** diastolic heart failure, heart failure with preserved ejection fraction, machine learning, artificial intelligence, computational biology, prognostic score, biomarkers

## Abstract

The ability to distinguish clinically meaningful subtypes of heart failure with preserved ejection fraction (HFpEF) has recently been examined by machine learning techniques but studies appear to have produced discordant results. The objective of this study is to synthesize the types of HFpEF by examining their features and relating them to phenotypes with adverse prognosis. A systematic search was conducted using the search terms “Diastolic Heart Failure” OR “heart failure with preserved ejection fraction” OR “heart failure with normal ejection fraction” OR “HFpEF” AND “machine learning” OR “artificial intelligence” OR 'computational biology'. Ten studies were identified and they varied in their prevalence of ten clinical variables: age, sex, body mass index (BMI) or obesity, hypertension, diabetes mellitus, coronary artery disease, atrial fibrillation, chronic kidney disease, chronic obstructive pulmonary disease or symptom severity (NYHA class or BNP). The clinical findings associated with the different phenotypes in > 85 % of studies were age, hypertension, atrial fibrillation, chronic kidney disease and worse symptoms severity; an adverse outcome was in 65 % to 85 % of studies identified diabetes mellitus and female sex and in less than 65 % of studies was body mass index or obesity, and coronary artery disease. COPD was a relevant factor in only 33 % of studies. Adverse clinical outcome - death or admission to hospital (for heart failure) defined phenogroups with the worst outcome. Combining the 4 studies that calculated the MAGGIC score showed a significant (p<0.05) linear relationship between MAGGIC score and outcome, using the one-year event rate. A new score based on strength of the evidence of the HFpEF studies analyzed here, using 9 variables (eliminating COPD), showed a significant (p<0.009) linear relationship with one-year event rate. Three studies examined biomarkers in detail and the ones most prominently related to outcome or consistently found in the studies were GDF15, FABP4, FGF23, sST2, renin and TNF. The dominant factors that identified phenotypes of HFpEF with adverse outcome were hypertension, atrial fibrillation, chronic kidney disease and worse symptoms severity. A new simplified score, based on clinical factors, was proposed to assess prognosis in HFpEF. Several biomarkers were consistently elevated in phenogroups with adverse outcomes and may indicate the underlying mechanism or pathophysiology specific for phenotypes with an adverse prognosis.

## Abbreviations

AF Atrial fibrillation

BIC Bayesian information criterion

BMI Body mass index

CAD Coronary artery disease

CKD Chronic kidney disease 

DM Diabetes mellitus

HF Heart failure

HFpEF Heart failure with preserved ejection fraction

LCA Latent class analysis

ML Machine learning

## Introduction

Heart failure with preserved ejection (HFpEF) which affects nearly half of all patients with heart failure is one of the major challenges in cardiology (Magana-Serrano et al., 2011[69]; Reddy and Borlaug, 2016[93]; Brouwers et al., 2013[10]; Gustafsson et al., 2003[40]; Owan et al., 2006[81]).) Not only does HFpEF have an extremely heterogeneous pathophysiology (Warbrick and Rabkin, 2019[128]; Mishra and Kass, 2021[73]), it has been resistant to most conventional therapies, which have been successful in the treatment of other kinds of heart failure (HF) (Shah, 2017[111]; Albakri, 2018[1]). The heterogeneity in pathophysiology has led investigators to examine whether there are different clinical entities that can be distinguished within the HFpEF patient population. This has been clinically challenging so that investigators have turned to data analytic techniques such as machine learning. Machine learning identifies patterns in complex data sets, which may exceed the human capacity to do so. Machine learning (ML) is being considered as the future of cardiovascular care as it can aid in understanding of a number of cardiovascular conditions (Giorgio et al., 2021[35]). Unsupervised ML seeks inherent patterns in large complex data sets (Hastie et al., 2009[42]) and the techniques of cluster analysis have proven to be reliable to distinguish subsets or groups (Huang, 1998[47]; Hastie et al., 2009[42]; Mushtaq et al., 2018[76]).

Investigators have applied machine learning strategies to their HFpEF patient populations and have found different phenogroups (Shah et al., 2015[112]; Hedman et al., 2020[43]; Nouraei and Rabkin, 2021[79]). Each study has defined different kinds of groupings and there has not been an attempt to synthesize this data and to gain a common understanding. The objective of this study is to evaluate and compare HFpEF phenotypes that have been recently proposed and to synthesis data in order to construct an easier approach to identify patient phenogroups with an adverse prognosis and a high-risk 'pathophysiology'.

## Methods

### Search strategy

**Figure 1:**
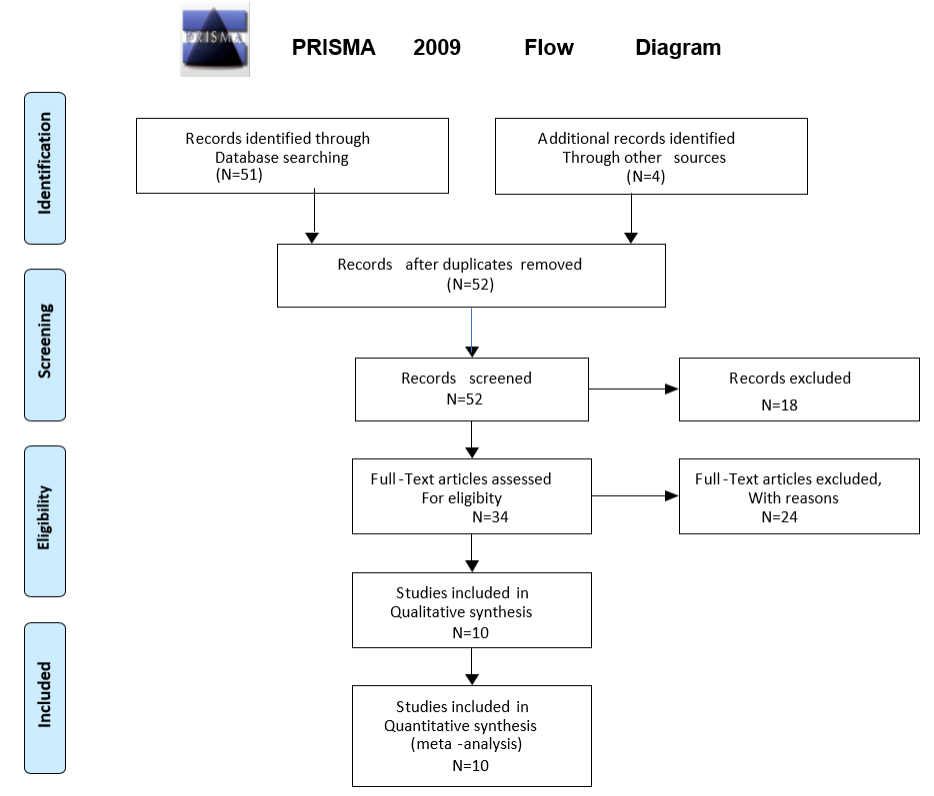
Outline of the search strategy using the PRISMA format

A systematic search was conducted through the Ovid Medline and Ovid Embase databases for the period prior to September 2021. The search was conducted according to the Preferred Reporting Items for Systematic Reviews and Meta-Analyses (PRISMA) format (Figure 1(Fig. 1)) (Moher et al., 2009[75]). The search terms applied were (“Diastolic Heart Failure” OR “heart failure with preserved ejection fraction” OR “heart failure with normal ejection fraction” OR “HFpEF”) AND “machine learning” OR “artificial intelligence” OR 'computational biology'. The pre-specified inclusion criteria were: (i) human subjects, (ii) English language publications. The exclusion criteria included (i) review articles, (ii) editorials or letters, (iii) unpublished papers, (iv) animal studies, (v) studies primarily on biomarkers or diagnostic testing e.g., echocardiography and clinical assessment only.

### Study selection

Using the search terms above, 51 total articles were identified and were combined with 4 additional records identified through other sources. An initial screening of titles and abstracts was conducted, followed by a screening of the remaining full-text articles (Figure 1(Fig. 1)). Articles were eliminated because of the exclusion criteria, leaving 10 articles that were included in qualitative and quantitative synthesis. Of the ten studies two utilized the same database and another two studies evaluated a modification of the same database. Most studies were eliminated because they applied machine learning to characterize biomarkers or diagnostic tests rather than utilizing machine learning to define phenotypes of HFpEF.

It is worth commenting on several excluded studies. Przewlocka-Kosmala et al. studied 177 patients with HFpEF and sought subtypes but on the basis of exercise stress testing data - an approach that other studies did not use (Przewlocka-Kosmala et al., 2019[87]). Tromp et al. subtyped patients with heart failure from 11 Asian regions using the ASIAN-HF registry of 6480 patients but the database included only 1204 patients with HFpEF so that the phenotypes identified were overwhelmingly influenced by the 80 % who did not have HFpEF (Tromp et al., 2018[122]). Sabbah et al. did not attempt to identify a new HFpEF clinical classification but rather to use ML analytic techniques to determine whether unique inflammation patterns exist in HFpEF and are associated with clinical severity or profibrotic state (Sabbah et al., 2020[99]). One study enrolled patients with echocardiographic evidence of diastolic dysfunction and evaluated whether they did or did not progress to have clinical evidence of heart failure (HFpEF) (Kaptein et al., 2020[53]). One study focused on comparing different ML models rather than trying to identify phenogroups (Angraal et al., 2020[4]). This is not an inclusive list of all studies excluded from analysis but rather a commentary on some of them.

## Results

### Methodologies

There were 10 studies that applied machine learning, artificial intelligence or computational biology to human data on HFpEF (Table 1(Tab. 1); References in Table 1: Casebeer et al., 2021[12]; Cohen et al., 2020[18]; Gu et al., 2021[37]; Hedman et al., 2020[43]; Kao et al., 2015[52]; Nouraei and Rabkin, 2021[79]; Schrub et al., 2020[107]; Segar et al., 2020[109]; Shah et al., 2015[112]; Woolley et al., 2021[131]). Nouraei and Rabkin examined 196 patients with HFpEF from an ambulatory clinic population and employed a non-hierarchical cluster analysis that used partitioning around medoids (PAM), on 47 variables (Nouraei and Rabkin, 2021[79]). The PAM approach assigns 'k' random entities to be medoids. The similarity between variables was assessed by the Gower distance that selects a particular distance metric which suits each variable type and scales the results to fall between 0 and 1 (Mushtaq et al., 2018[76]). A silhouette plot, an aggregated measure of similarity between observations within a cluster compared to observations in neighboring clusters determined the optimal number of the number of clusters (Kaufman and Rousseeuw, 1990[55]). The choice of the number of clusters was based on the largest silhouette width, which is an index reflecting the compactness of clusters and their separation from each other (Kaufman and Rousseeuw, 1990[55]). 

Wooley et al. performed an unsupervised cluster analysis using 363 markers from 429 patients with HFpEF from the Scottish cohort of BIOSTAT-CHF study which had data on 1738 patients from six centers in Scotland, previously admitted with HF requiring diuretic treatment (Woolley et al., 2021[131]). Patients with LVEF greater or equal to 50 % were evaluated. Principle component analysis was performed in order to reduce biomarker dimensions and collinearity. Clustering was performed on principle components with an eigenvalue of one or above using a hierarchical clustering algorithm (Woolley et al., 2021[131]).

Casebeer et al. (2021[12]) identified 1515 patients newly diagnosed with heart failure and an ejection fraction of 50 % or greater who were enrolled in a US Medicare Advantage Prescription Drug healthcare. Hierarchical clustering assigned each observation to their own cluster and then iteratively merges clusters based on their distance to other clusters. A high-performance clustering procedure was used to perform the cluster analysis. For categorical variables, the Hamming method was used to create a distance matrix on the reduced set of baseline characteristics, and then the Ward method was used to apply hierarchical clustering to the distance matrix data source (Casebeer et al., 2021[12]).

Gu et al. (2021[37]) recruited a longitudinal cohort study of adults with HF from Shanghai Ninth People's Hospital. HFpEF was defined by clinical features of HF with LVEF greater than or equal to 50 %. Recruitment occurred where the patient was either in the hospital for a primary diagnosis of HFpEF (the assessment was performed following stabilization of the acute HF) or in the outpatient setting within 3 months of an episode of decompensated HF (requiring hospitalization or treatment in an outpatient setting) (Gu et al., 2021[37]). Exclusion criteria included severe valve disease, transient acute pulmonary edema in the context of primary acute coronary syndrome, end-stage renal failure (estimated glomerular filtration rate). Hierarchical clustering utilized 11 prospectively selected features: age, gender, body mass index (BMI), AF, hypertension, ischemic heart disease (CAD), type 2 DM, estimated glomerular filtration rate (eGFR), hemoglobin, E/e' ratio on echocardiogram and BNP. Model-based clustering of standardized variables was performed in R using the Mclust function in the mclust package, with default settings, and the optimal model and number of clusters determined by the maximum BIC (Gu et al., 2021[37]).

Hedman et al. (2020[43]) and Schrub et al. (2020[107]) used cluster analytic techniques on the Karolinska-Rennes cohort (KaRen study) (Donal et al., 2009[25]). The KaRen study included patients who presented to the Emergency with the clinical signs and symptoms of HF, fulfilling the Framingham criteria, with an elevated BNP or N-terminal prohormone of BNP, with an echocardiographic measurement of LVEF greater than 45 % within the first 72 hours of presentation (Donal et al., 2009[25]). Hedman et al. (2020[43]) applied data analytics to a model based on data from 320 patients collected in stable condition with HFpEF clustering 32 echocardiographic and 11 clinical or laboratory variables from 320 HFpEF outpatients in the collected in stable condition in the KaRen study cohort. Model-based clustering of standardized variables performed used the Mclust function and the optimal model and number of clusters was determined by the maximum BIC with three multinomial classification methods (Elastic Net, Neural Networks and Naive Bayes, in the R statistical package (Hedman et al., 2020[43]). 

Schrub et al. (2020[107]) performed a cluster analysis of 538 patients from the KaRen study. A hierarchical cluster analysis was conducted using two-stage density linkage, specifying five neighbors for k-nearest neighbor density estimation (Schrub et al., 2020[107]).

Segar et al. (2020[109]) and Cohen et al. (2020[18]) examined data from the TOPCAT study, a large multicenter international trial evaluating the efficacy of spironolactone therapy in patients older than 50 years of age with symptomatic HFpEF and an LVEF ≥ 45 %. Latent class analysis (LCA) was used to determine clusters of clinical phenotypes. LCA uses finite mixture modeling to classify individuals into mutually exclusive subgroups, maximizing within-group similarities and between-group differences on the basis of multiple observed population characteristics (Cohen et al., 2020[18]). To determine the optimal number of phenogroups, several metrics were utilized, specifically the parametric bootstrap likelihood ratio test, Akaike's Information Criterion, BIC, and sample-size-adjusted BIC (Cohen et al., 2020[18]).

Segar et al. (2020[109]) examined a subset of the TOPCAT participants with echocardiographic data (n = 654). A total of 61 continuous and categorical variables were used that encompassed a range of domains including demographics, clinical variables, laboratory data, electrocardiographic characteristics, and echocardiographic factors (Segar et al., 2020[109]). To determine the optimal number of phenogroups, they used model-based clustering with optimization of the BIC and Dunn index (Segar et al., 2020[109]). The BIC introduces a penalty term for the number of parameters in the model thus selecting models with better fit, while the Dunn index identifies clusters that are compact, with small intra-cluster variance, and well separated, where the centers of each cluster are far apart (Segar et al., 2020[109]). They selected the optimal number of clusters choosing the model with the lowest absolute value of the BIC and the highest Dunn index.

Shah et al. (2015[112]) prospectively collected data on 397 patients with HFpEF and performed detailed clinical, laboratory, ECG, and echocardiographic phenotyping of patients who comprised consecutive patients who were recruited after heart failure hospitalization. Agglomerative hierarchical clustering was used and hierarchical clustering was performed with the 'hclust function (in R 3.0.1)', with the dissimilarity matrix given by euclidean distance and the average linkage score used to join similar clusters (Shah et al., 2015[112]). BIC was used to penalize increases in model complexity such as a greater number of clusters or variability in standard deviation across variables and across clusters (Shah et al., 2015[112]).

Kao et al. (2015[52]) examined data from the I-PRESERVE study that enrolled HFpEF patients ≥ 60 years old with New York Heart Association (NYHA) class II-IV symptoms and hospitalization owing to heart failure or NYHA class III-IV symptoms and pulmonary congestion on X-ray. Patients were characterized according to 11 prospectively selected clinical features: LCA was performed using the polkas library in the R statistical package. LCA definitions were derived using maximum-likelihood estimation to identify the most common patterns. The optimal number of subgroups for I-PRESERVE was determined using the first minima of the BIC and χ2 statistic. Probabilities of membership in each subgroup for every LCA variable were used to determine the most likely subgroup for each patient (Kao et al., 2015[52]).

### Phenogroups

Of the ten studies three suggested that there were six phenotypes and six studies suggest that there were only three phenotype (Table 2(Tab. 2); References in Table 2: Casebeer et al., 2021[12]; Cohen et al., 2020[18]; Gu et al., 2021[37]; Hedman et al., 2020[43]; Kao et al., 2015[52]; Nouraei and Rabkin, 2021[79]; Schrub et al., 2020[107]; Segar et al., 2020[109]; Shah et al., 2015[112]; Woolley et al., 2021[131]). One study reported that the 'optimal number of clusters was six', but due to the small size of two clusters (*n* = 3 and *n* = 2), they focused on the (remaining four) patient clusters (Woolley et al., 2021[131]). It is noteworthy that in the KaRen cohort, one evaluation considers that there were six phenotypes while another analysis concluded that there were only three phenotypes. The clinical characteristics of each phenotype are discussed.

Nouraei and Rabkin (2021[79]) found six significantly different phenotypes or clusters. **Phenogroup 1 **women with a low proportion of vascular risk factors (HFpEF_1_). **Phenogroup 2 **men with a high proportion of coronary artery disease (CAD), dyslipidemia, higher serum creatinine, and diastolic dysfunction (HFpEF_2_). **Phenogroup 3 **women with a high proportion of hypertension and diabetes, but lower proportion of kidney disease and diastolic dysfunction (HFpEF_3_). **Phenogroup 4 **older women with high rates of atrial fibrillation (AF), chronic kidney disease (HFpEF_4_). **Phenogroup 5 **men with the highest BMI, and high proportion of CAD, obstructive sleep apnea, and poorly controlled diabetes (HFpEF_5_). **Phenogroup 6 **men with high rates of atrial fibrillation (AF), elevated BNP, biventricular remodeling (HFpEF_6_) (Nouraei and Rabkin, 2021[79]).

Woolley et al. (2021[131]) reported four distinct patient phenotypes. Patients in **Phenogroup 1 **had the highest prevalence of chronic kidney disease (CKD, 74 %) and diabetes mellitus (53 %). Patients in **Phenogroup** **2** had the oldest age and frequency of age-related morbidities such as AF (47 %) and hypertension (72 %), however these did not reach significance. Patients in P**henogroup** **3** had the youngest age, largest body size, least symptoms and lowest N-terminal pro-B-type natriuretic peptide (NT-proBNP) levels. Patients in **Phenogroup** **3** were youngest (mean age 74 years), had the lowest prevalence of most comorbidities, except obesity (mean body mass index 30.4 kg/m^2^), were the least symptomatic and had the lowest plasma NT-proBNP). Patients in **Phenogroup** **4** had the highest prevalence of ischemic etiology, smoking and chronic lung disease, the most symptoms, as well as highest NT-proBNP and troponin levels (Woolley et al., 2021[131]). 

Casebeer et al. (2021[12]) identified three different (clusters) phenotypes. **Phenogroup 1 **patients had the lowest prevalence of heart failure comorbidities and highest mean age; **Phenogroup 2** patients had higher prevalence of metabolic syndrome and pulmonary disease, despite younger mean age. **Phenogroup 3 **patients had higher prevalence of cardiac arrhythmia and renal disease (Casebeer et al., 2021[12]). 

Gu et al. (2021[37]) identified three different (clusters) phenotypes. **Phenogroup 1 **was composed of younger individuals (69 years) with relatively good New York Heart Association class (NYHA), preserved renal function and a lower prevalence of T2DM (30.0 %) and IHD (33.6 %), and higher level of hemoglobin (Gu et al., 2021[37]). **Phenogroup 2** was characterized by older age (71 years), higher proportion of women (50.0 %) and higher prevalence of AF (46.7 %). **Phenogroup 3** had an intermediate age (70 years), with higher BMI (25.0), the higher prevalence of CAD (47.6 %) and T2DM (39.4 %), and severe HF symptoms assessed by NYHA. BNP level was highest in **Phenogroup 3** and lowest in **Phenogroup 1**. All 3 phenotype groups had similar rates of beta-blockers or spironolactone treatment but ACEI/ARB prescription was much more common in **Phenogroup 3** (Gu et al., 2021[37]).

Hedman et al. (2020[43]) identified six composite phenogroups. **Phenogroup 1** was younger, had more cardiovascular risk factors and progressed to CKD (67 %). **Phenogroup 2** had more severe HF, with the greatest degree of diastolic dysfunction (at least 30 % of patients with a grade II or higher) and the worst right ventricular function and high prevalence of COPD (30 %). **Phenogroup 3** had mild HF and HF symptoms, but the prevalence of obesity was 48 %. **Phenogroup** **4** were male but otherwise similar to the female groups 5 and 6, with hypertension (75 %), left atrial enlargement and AF (90 %). This group had the largest proportion of pacemakers (25 %) and previous myocardial infarction (21 %), suggesting an ischemic etiology of HF. **Phenogroups 5 **and** 6** were older women with a high proportion of hypertension (80 %) and AF (96 %) and lower body mass index (BMI) (mean=27), and CAD (40 %) (Hedman et al., 2020[43]). 

Schrub et al. (2020[107]) performed a cluster analysis of 538 patients from the KaRen study and identified 356 'analyzable' patients (mean age 76 years; 44 % men). **Phenogroup 1** (n = 128) comprised overweight, relatively young men, in sinus rhythm, with reduced renal function. **Phenogroup 2** (n = 134) comprised women, most of whom were reported to have 'conserved left ventricular function'. **Phenogroup 3 **(n = 94) had the highest incidence of mitral regurgitation, atrial remodeling and rhythm disorders (Schrub et al., 2020[107]).

In TOPCAT study participants, Segar et al. (2020[109]) concluded that there were three phenotypes. **Phenogroup 1** had significantly higher BMI, more severe HF symptoms as assessed by New York Heart Association class, and higher burden of DM, dyslipidemia, and atherosclerotic cardiovascular disease as compared with the other groups. **Phenogroup 1** participants had lower hemoglobin levels and higher blood glucose, creatinine, and blood urea nitrogen levels as compared with the other groups. It had the highest BNP levels. **Phenogroup 2 **had the lowest burden of DM, HF symptoms, and atherosclerotic cardiovascular disease. Liver function test abnormalities were more common in **Phenogroup 2** compared to other groups. **Phenogroup 3** had the lowest BMI and an intermediate burden of atherosclerotic vascular disease and DM as compared with the other groups. BNP was the lowest of all three phenogroups. Blood glucose levels, renal function parameters, hemoglobin levels were comparable in **Phenogroups 2 and 3**. 

The phenogroup algorithms from TOPCAT were applied to the data from the RELAX trial on 198 participants (Segar et al., 2020[109]). Similar to the TOPCAT derivation cohort, **Phenogroup 1** in the RELAX validation cohort had the highest burden of DM, higher BMI and BNP levels, and higher left atrial size and tricuspid regurgitation. **Phenogroup 2** had the lowest prevalence of cardio metabolic disease but higher burden of diastolic dysfunction (Segar et al., 2020[109]). **Phenogroup 3** participants had the lowest BMI, renal dysfunction, and BNP levels (Segar et al., 2020[109]).

Cohen et al. (2020[18]) also examined the TOPCAT study participants. **Phenogroup 1** was composed of younger individuals (mean age 61 years) with relatively preserved functional class, the highest prevalence of smoking (24 %) among the groups, along with relatively preserved renal function and a low prevalence of diabetes (9 %) (Cohen et al., 2020[18]). **Phenogroup 2** was characterized by older age (mean age 77 years), the highest proportion of women (56 %), a high prevalence of atrial fibrillation (49 %) and CKD but a low prevalence of diabetes and obesity. **Phenogroup 3** exhibited intermediate age (mean age 66 years), with a very high prevalence of obesity (98 %), DM (88 %), and impaired functional class. It also had a high prevalence of CKD (57 %) (Cohen et al., 2020[18]). 

Shah et al. (2015[112]) identified three phenogroups that were significantly different from each other. **Phenogroup 1** was younger and had lower BNP than participants in the other groups. **Phenogroup 1** had the least electric and myocardial remodeling and dysfunction and the least hemodynamic derangement, although even in this group, 65 % had at least moderate (grade 2) diastolic dysfunction, and the mean pulmonary artery systolic pressure was 42 mm Hg. **Phenogroup 2** had the highest prevalence of obesity, DM, obstructive sleep apnea, worst LV relaxation, highest pulmonary capillary wedge pressure, and highest pulmonary vascular resistance. **Phenogroup 3** was the oldest, was most likely to have chronic kidney disease and had the highest BNP. **Phenogroup **3 had the most severe electric and myocardial remodeling with the longest QRS duration, largest QRS-T angle, highest relative wall thickness and LV mass index, highest E/e' ratio, and worst RV function (Shah et al., 2015[112]). 

Kao et al. (2015[52]) reported on the analysis of the IPRESERVE study and identified 6 phenogroups. **Phenogroup 1** (A) (median age 65 years) was 100 % men. **Phenogroup 2 (**B) (median 65 years) was 96 % women. **Phenogroups 1 **(A) and 2 (B) had low rates of AF, renal dysfunction, and valvular disease. **Phenogroup 3** (C) (median 70 years) had high rates of obesity, DM, hyperlipidemia, CAD, and anemia with worse renal function than other subgroups. **Phenogroup 4** (D) (median 73 years) had 100 % women with average rates of DM, hyperlipidemia, and obesity and renal insufficiency. **Phenogroup 5 (**E) (median 75 years) was 100 % men with lower BMI, excess AF, and CAD. **Phenogroup 6 (**F) was predominantly women (78 %) of advanced age (median 82 years) with lower BMI and high rates of AF, valvular disease, renal dysfunction, and anemia. This group had the highest NT-proBNP (Kao et al., 2015[52]).

### Comparison of clinical characteristics

There were differences in clinical features of the phenotypes between the studies (Table 2(Tab. 2)). Age was available in all studies. It was a significant factor in 8, not significant in one (Segar et al., 2020[109]) and not analyzed in one study (Kao et al., 2015[52]). Most studies showed that age was significantly different between the phenotypes. Sex was examined in all studies and was a significant factor in 8, although barely significant at the 5 % level in one study (Shah et al., 2015[112]). Two studies did not find a difference in the sex distribution of individuals between phenotypes (Segar et al., 2020[109]; Woolley et al., 2021[131]). Body mass index or overweight status was available in all 10 studies and was significantly different between phenotypes in 6 of those studies and not analyzed in one. Hypertension was reported in 9 of the studies and was significantly different between phenotypes in 5 studies. Atrial fibrillation was reported in 9 studies and was significantly different across phenogroups in 8 studies, not significant in one. DM was available in all 10 studies and was significantly different between phenotypes in 9 studies, although in one study it was barely significant at the 5 % level. CAD was available in 8 studies and was significantly different between phenotypes in 5 studies. CKD or eGFR data was available in all 10 studies and was significantly different between phenotypes in all 10 studies. COPD was available in 9 studies and there were significant differences across phenotypes as only 3 studies reported a relationship so that the majority (66.6 %) of studies did not find a difference in COPD amongst phenotypes. Heart failure severity was assessed by NYHA class (Class III or greater) or by the degree of BNP or NT BNP elevation and was available in 8 studies. There were significant differences across phenotypes in 7 of those studies and it was not analyzed in one study. Symptomatic heart failure severity likely mirrors the severity of diastolic dysfunction.

## Outcome

**Figure 2:**
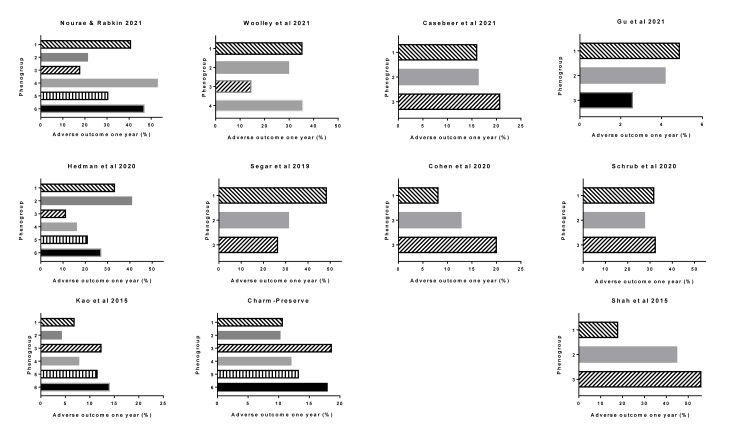
One year outcome in each phenogroup in each of the studies cited. The Charm-Preserve study was included in one of the publications.

Most of the studies examined the outcome of each of their phenogroups. The duration of follow-up varied between the studies. A standardizing of follow-up time of one-year (12 month) time was selected. Data were taken from the tables or graphical extrapolations for each publication. Although there were differences in the type of outcomes between studies. Most studies used mortality and (re-)admission to hospital. Repeat hospitalizations was usually defined as readmission for heart failure but in some it was not specified. Nevertheless, it is a reasonable assumption that in a heart failure population, hospital readmission for cardiac causes would most likely be heart failure. When the data came from a clinical trial, the primary end point was used which was usually death or hospitalization (Table 1(Tab. 1)). The relationship to the type of phenotype was identified. In general, there were significant differences in outcomes for most of the studies according to the HFpEF phenotype (Figure 2(Fig. 2); References in Figure 2: Casebeer et al., 2021[12]; Cohen et al., 2020[18]; Hedman et al., 2020[43]; Kao et al., 2015[52]; Nouraei and Rabkin, 2021[79]; Segar et al., 2020[109]; Woolley et al., 2021[131]). It showed that overall phenotyping HFpEF identified groups with different outcomes. The differences, however, varied considerably between the studies, in part because of the different types of subgroups and also likely different nature of the populations. 

Nouraei and Rabkin reported that the worst one year outcome was in **Phenotype 4**, followed by 6 and then 1, with the best outcome being in group 3 (Nouraei and Rabkin, 2021[79]). 

Wooley et al. (2021[131]) reported that after a median follow-up of 21 months, the occurrence of death or HF hospitalization was “highest in **Phenogroups 1** and **4 **(62.1 % and 62.8 %, respectively) and lowest in cluster 3 (25.6 %)'. 'Rate of HF hospitalization alone' was highest in cluster 1 (36 %), compared with 23 % in cluster 2, 18 % in cluster 3, and 21 % in cluster 4 (Woolley et al., 2021[131]). 'After correction for age, sex, previous HF hospitalization and NYHA class, compared to cluster 1, patients in clusters 2 and 3 had a lower risk of death or HF hospitalization' [hazard ratio 0.58 and 0.30 respectively] (Woolley et al., 2021[131]).

Casebeer et al. (2021[12]) reported that their phenotype 3 had the highest 1-year heart failure related hospitalization rates. 

Gu et al. (2021[37]) reported on the primary outcome defined as all-cause mortality and the secondary outcome which was the composite endpoints of death or HF hospitalization. The 1 year and 5 year mortality data for all-cause mortality was highest in **Phenogroup 3 **followed by** Phenogroup 2 **and** Phenogroup 1**. Similar patterns of association were also noted between phenogroups and composite endpoints, with a graded decrease in the incidence of all-cause mortality or HF hospitalization from **Phenogroup 3, Phenogroup 2 **to** Phenogroup 1**. In multivariable adjusted Cox models, HFpEF phenogroup was an independent risk factor for all-cause mortality or composite endpoints (Gu et al., 2021[37]).

In the KaRen cohort, the initial analysis by Schrub et al. (2020[107]) identified three phenogroups. They reported 'no statistical difference between the three clusters for the primary endpoint'. When death was the only criterion analyzed, **Phenogroup 3** (cluster 3) showed higher rates of short and midterm mortality (Schrub et al., 2020[107]). In the same population, Hedman et al. (2020[43]) did a more detailed analysis of a smaller sample of the same cohort and identified 6 phenotypes. They reported that 'the composite end point was significantly different (log rank p<0.001) between the phenogroups to the 18 months follow-up, with **Phenogroup 2** having the highest and **Phenogroup 3** having the lowest event rates'. This phenogroup also had high NT-proBNP levels and the highest proportion of patients in New York Heart Association class IV.

In the TOPCAT trial, the primary outcome was a composite of hospitalization for management of HF, CV death or aborted cardiac arrest (Segar et al., 2020[109]). Cohen et al. found that in their analysis of TOPCAT that **Phenogroup 3** had the worst outcome followed by **Phenogroup 2 **with** Phenogroup **1 having the best outcome (Cohen et al., 2020[18]). In the more detailed analysis by Segar et al. 'the cumulative incidence of the primary composite outcome was highest in **Phenogroup 1** followed by **Phenogroup** **2** and **3** (48.5 %, 31.5 %, and 26.6 %, respectively)'. Similar patterns of association were also noted between phenogroups and non-fatal hospitalization outcomes, with a graded decrease in the incidence of all-cause and HF hospitalization from** Phenogroup 1 **to** Phenogroup 3**. The cumulative incidence of all-cause mortality was significantly higher in P**henogroup 1** and comparable between **Phenogroups 2 **and** 3**. In contrast, the cumulative incidence of major atherosclerotic CV event was significantly higher in **Phenogroup 1**, but **Phenogroup 2** had a lower risk compared to **Phenogroup **3 (Segar et al., 2020[109]). 

The phenogroup algorithms from TOPCAT were applied to the data from the RELAX trial. There was a statistically significant difference in the clinical status rank on follow-up between phenogroups. **Phenogroup 1** in the RELAX trial cohort, as in the TOPCAT cohort, had significantly worse clinical status rank on follow-up as compared with the other two groups (Segar et al., 2020[109])

Shah et al. (2015[112]) identified three phenogroups with **Phenogroup 3** having the worst outcome and **Phenogroup 1** having the best outcome.

In I Preserve, the primary outcome was all-cause mortality or hospitalization for a cardiovascular reason. The secondary outcome was HF hospitalization or death due to either HF or sudden death. Mean follow-up was 49.5 months. **Phenogroup 6** and **3** (F and C) had the highest rate of both outcomes whereas **Phenogroup 2** (B) had the lowest primary outcome (Kao et al., 2015[52]). Using the algorithms from I Preserve and applying it to the CHARM study, mean follow-up were 36.6 months and where the primary endpoint was a composite of cardiovascular death or HF hospitalization. The results were similar with the worst prognosis in **Phenogroup 6** and **3** (F and C) with the best outcome in **Phenogroup 2** (B) (Kao et al., 2015[52]). 

The approach developed using the I Preserve data was applied to the CHARM-preserve study. A similar pattern of differences between the phenogroups was observed although the magnitude of the absolute adverse outcome was somewhat higher in CHARM-Preserve.

### The clinical characteristics of phenotypes with the worst outcome

Focusing on the clinical characteristics of the phenogroup from each study that had the worst prognosis revealed different compositions of the phenogroup with the worst outcome between groups.

Nouraei and Rabkin (2021[79]) reported that the worst outcome was in **Phenogroup 4 **that were predominantly older women with high rates of atrial fibrillation and chronic kidney disease (HFpEF_4_).

Woolley et al. (2021[131]) reported that the worst outcome was in **Phenogroup 1 **that had the highest prevalence of chronic kidney disease (CKD, 74 %) and diabetes mellitus (53 %). The next worst outcome, which was similar, was in **Phenogroup** **4, **which had the highest prevalence of ischemic etiology, smoking and chronic lung disease, the most symptoms, as well as highest NT-proBNP and troponin levels (Woolley et al., 2021[131]). 

Casebeer et al. observed the worst outcome in **Phenogroup 3** patients that had the higher prevalence of chronic kidney disease and cardiac arrhythmia (Casebeer et al., 2021[12]). 

Gu et al. (2021[37]) found the worst outcome in **Phenogroup 3**. Patients in this group were intermediate in age (70 years), with higher BMI, the higher prevalence of CAD and T2DM and most severe HF symptoms assessed by NYHA and highest BNP.

Hedman et al. (2020[43]) reported that their **Phenogroup 2** had more severe HF, with the greatest degree of diastolic dysfunction and the worst right ventricular function and a high prevalence. 

In TOPCAT study participants, Segar et al. found the worst outcome in **Phenogroup 1** that had significantly higher BMI, more severe HF symptoms as assessed by New York Heart Association class, higher burden of DM, dyslipidemia, and atherosclerotic cardiovascular disease as compared with the other phenogroups (Segar et al., 2020[109]). **Phenogroup 1** individuals had the highest BNP levels.

The phenogroup algorithms from TOPCAT were applied to the data from the RELAX trial on 198 participants (Segar et al., 2020[109]). Similar to the TOPCAT derivation cohort, **Phenogroup 1** in the RELAX validation cohort had the highest burden of DM, higher BMI and BNP levels, and larger left atrial size. 

Cohen et al. (2020[18]) also examined the TOPCAT study participants and identified **Phenogroup 3** that exhibited intermediate age (mean age 66 years), with a very high prevalence of obesity (98 %), DM (88 %), and impaired functional class. It also had a high prevalence of CKD (57 %). 

Shah et al. (2015[112]) analysis found that **Phenogroup 3** was the oldest, was most likely to have CKD and had the highest BNP. **Phenogroup 3** also had the most severe electric and myocardial remodeling with the longest QRS duration, largest QRS-T angle, highest relative wall thickness and LV mass index, highest E/e' ratio, and worst RV function. 

Kao et al. (2015[52]) found that **Phenogroup 3** (C) (median 70 years) had high rates of obesity, DM, hyperlipidemia, CAD, and worse renal function than other subgroups. **Phenogroup 6 (**F) was predominantly women (78 %) of advanced age (median 82 years) with lower BMI and high rates of AF, valvular disease, and CKD. This group had the highest NT-proBNP. The phenogroup algorithms from I Preserve were applied to the data from the CHARM-Preserve trial. The CHARM-Preserve study enrolled adults with an ejection fraction *> *40 %, NYHA class II-IV symptoms, for ≥ 4 weeks and a history of HF hospitalization and randomized them to candesartan or placebo. The primary endpoint was a composite of cardiovascular death or HF hospitalization. In that study, **Phenogroup 3** had the worst outcome which validated the I Preserve algorithm. 

### Biomarker characterization of high risk HFpEF phenotypes

There were only three studies that examined biomarkers in detail in their patient subgroups (Table 3(Tab. 3); References in Table 3: Cohen et al., 2020[18]; Hedman et al., 2020[43]; Woolley et al., 2021[131]). Woolley et al. reported that in their **Phenogroup** **1**, there were 29 proteins that were significantly up-regulated compared to the rest of the clusters (Woolley et al., 2021[131]). These included significant biological processes - members of the tumor necrosis factor (TNF) family and their receptors. In **Phenogroup 2,** no proteins were found to be significantly up- or down-regulated. In **Phenogroup** **3,** a total of 26 proteins were significantly down-regulated and there was a significant association with the TNF family and its receptors as well as some cytokine and cytokine receptors. In **Phenogroup** **4**, thirty-four proteins were found to be significantly up-regulated and one protein was significantly down-regulated. The up-regulated proteins were considered to be associated with six biological processes 'protein serine/threonine kinase inhibitor activity, regulation of receptor internalization, viral myocarditis, Kaposi sarcoma-associated herpes virus infection, PI3K/AKT signaling in cancer and positive regulation of phosphatidylinositol 3-kinase activity (Woolley et al., 2021[131]).

In a subset of Swedish KaRen patients Hedman et al. examined plasma protein data and reported ten plasma proteins were directly associated with phenotypical variables and comorbidities (Hedman et al., 2020[43]). Specific soluble ST2 (sST2) was highest in **Phenogroup 2**, that had the worst outcome, while **Phenogroup 1** had the highest levels of proteins associated with CKD and HF incidence (FGF23, PlGF, TRAIL-R2, U-PAR) (Hedman et al., 2020[43]). 

The TOPCAT study, assessed 49 biomarkers and presented the level of significance after correction for multiple comparisons (Cohen et al., 2020[18]). Ten serum proteins were significantly different across their three phenogroups after adjusting for multiple comparisons of the initial analysis of the 49 biomarkers. The biomarkers were considered to fall into one of several large groups: one group included biomarkers of fibrosis/tissue remodeling, inflammation, renal injury/dysfunction, and liver fibrosis. Inflammatory biomarkers included TNF and its receptor family (Cohen et al., 2020[18]). Other clusters were composed of neurohormonal regulators of mineral metabolism, intermediary metabolism and adipocyte biology (fatty acid binding protein-4 and growth differentiation factor-15), angiopoietin-2 (related to angiogenesis), matrix metalloproteinase (related to extracellular matrix turnover) (Cohen et al., 2020[18]). sST-2 was significant although not after adjusting for multiple comparisons (Cohen et al., 2020[18]). 

A combination of biomarkers was strongly predictive of the probability of an adverse outcome and markedly improved the risk prediction when added to the MAGGIC risk score (Chirinos et al., 2020[14]). In an independent cohort, the model strongly predicted the risk of an adverse outcome, which was also independent of the MAGGIC risk score (Chirinos et al., 2020[14]).

Several proteins are worth discussing in detail because they were strongly associated with a phenotype with an adverse prognosis or were identified in two different studies specifically GDF15, FABP4, FGF23, sST2, matrix metalloproteinases and their inhibitors, and TNF receptors.

### Growth differentiation factor-15 

Two of the three studies identified high levels of Growth Differentiation Factor-15 (GDF-15), in the phenotype with the worst prognosis. We have previously reviewed GDF-15 in HFpEF (Rabkin and Tang, 2021[91]). GDF-15 is secreted as a pro-peptide that is cleaved in the endoplasmic reticulum, to an active peptide, then it can enter the circulation (Fairlie et al., 2001[28]; Unsicker et al., 2013[124]). GDF-15 mRNA and pro-peptide expression is induced through nitric oxide-peroxynitrite-dependent signaling pathways in the heart where it can function in a protective capacity against myocardial ischemia (Kempf et al., 2006[56]). The pro-survival effects of GDF-15 are medicated in part through a phosphoinositide3-OH kinase pathway (Kempf et al., 2006[56]). Over-expression of GDF-15 reduces expression of phosphorylated RelA p65, pro-inflammatory and pro-apoptotic genes and increased Foxo3, phosphorylation (Zhang et al., 2017[133]). These data, in conjunction with the findings using recombinant GDF-15, suggest that GDF-15 limits myocardial tissue damage and apoptosis (Kempf et al., 2006[56]; Zhang et al., 2017[133]). GDF-15 also enhances hypertrophic cardiomyocyte cell growth (Heger et al., 2010[44]). Hypertrophic signaling is mediated via the kinases PI3K and ERK and the transcription factor R-SMAD1 (Heger et al., 2010[44]). Previously, higher circulating levels of GDF-15 have been associated with a worse long term prognosis in acute heart failure or in HFrEF (Lin et al., 2014[66]; Cotter et al., 2015[19]; Bettencourt et al., 2018[8]). Circulating GDF-15 levels are higher in patients with HFpEF compared to controls (Stahrenberg et al., 2010[115]; Dinh et al., 2011[23]; Santhanakrishnan et al., 2012[105]; Sinning et al., 2017[114]). A meta-analysis of those studies found that compared to a control group, there were significantly and consistently higher level of GDF-15 in HFpEF (Rabkin and Tang, 2021[91]). 

The magnitude of GDF-15 elevation relates to the alteration in left ventricular diastolic dysfunction identified on echocardiography (Stahrenberg et al., 2010[115]; Dinh et al., 2011[23]; Santhanakrishnan et al., 2012[105])*.* Meta-analysis revealed that the greater the elevation of circulating GDF-15 correlates with the greater degree of diastolic dysfunction (Rabkin and Tang, 2021[91]). Some data suggest that BNP does not add meaningful diagnostic information after considering GDF-15 (Santhanakrishnan et al., 2012[105])*.*

There is a slightly higher level of GDF-15 in patients with HFpEF compared to HFrEF but it is often not statistically significant (Rabkin and Tang, 2021[91]). Whether this reflects the association of diastolic dysfunction in some cases of systolic dysfunction is unknown.

Two mechanisms that can produce an increase in left ventricular end diastolic pressures and reduce left ventricular diastolic compliance are increased left ventricular mass and cardiac fibrosis (Wesseling et al., 2020[130]). Pro-hypertrophic and anti-hypertrophic effects of GDF15 have been described, suggesting effect of GDF-15 in cardiac hypertrophic responses dependent on the environmental circumstances (Wesseling et al., 2020[130]).

Up-regulated expression of GDF-15, in newborn rat cardiac fibroblasts after transfection with Gdf15 increased cell proliferation rate and expression of fibrosis markers (Col1α and αSMA) (Guo et al., 2021[39]), while down-regulation of GDF-15 inhibited cardiac fibrosis (Guo et al., 2021[39]) through the MAPK/ERK1/2 pathway. GDF-15 could induce cell proliferation through the PI3K/Akt and ERK signaling pathways (Jin et al., 2012[50]). Circulating GDF-15 was significantly correlated with the amount of myocardial fibrosis in end-stage HF patients prior to LVAD implantation (Lok et al., 2012[68]). However, anti-fibrotic and pro-fibrotic effects of GDF15 have been described (Wesseling et al., 2020[130]) so that the causative role of GDF15 in HFpEF requires further investigation.

In morbidly obese individuals, GDF-15 levels appear to correlate better with diastolic dysfunction than NT-proBNP levels so that GDF-15, adds incremental value to NT-proBNP (Baessler et al., 2012[6]).

### FGF23

Fibroblast growth factor-23 (FGF23), produced mainly by osteoblasts/osteocytes, functions to inhibit renal tubular phosphate reabsorption and regulates plasma phosphate levels (Martin et al., 2012[70]). FGF23 is also expressed in the heart, and is markedly enhanced in settings of cardiac remodeling and heart failure (Leifheit-Nestler and Haffner, 2018[63]). FGF23 promotes hypertrophic growth of cardiac myocytes acting through FGF receptor-4 dependent activation of phospholipase Cγ/calcineurin/nuclear factor of activated T cell signaling independent of its co-receptor klotho (Leifheit-Nestler and Haffner, 2018[63]). FGF23 is expressed in cardiac myocytes, cardiac fibroblasts, vascular smooth muscle and endothelial cells in coronary arteries, and in inflammatory macrophages (Leifheit-Nestler and Haffner, 2018[63]). Because of this diversity in cellular expression, FGF23 can stimulate cardiac hypertrophy and/or cardiac fibrosis dependent in part on cardiac status and other factors (Leifheit-Nestler et al., 2021[64]).

In the MESA cohort of 6542 persons who were free of cardiovascular disease at baseline, FGF23, even after adjusting for other factors, was association with the incidence of HFpEF (Almahmoud et al., 2018[3]). In small clinical studies, circulating levels of another FGF, FGF21, correlate with echocardiographic parameters of diastolic function and LV end-diastolic pressure (Chou et al., 2016[16]). In patients with HFpEF, higher FGF23 levels are independently associated with decreased exercise capacity (Ghuman et al., 2021[34]). Higher circulating FGF23 levels are associated with more cardiac fibrosis estimated by cardiac magnetic resonance (Roy et al., 2020[96]). 

In summary FGF23 is likely a causal factor in production of HFpEF because it fulfills criteria for causality (Rabkin and Sackett, 1982[90]) being able to predict the development of the condition, correlates with the severity of the condition and has a reasonable biologic mechanism, specifically FGF23 can stimulate cardiac hypertrophy and/or cardiac fibrosis which will produce diastolic dysfunction.

### Fatty acid-binding protein 4 

Fatty acid-binding proteins (FABPs) comprise a family of intracellular lipid chaperones that regulate lipid trafficking and responses in cells and are linked to metabolic and inflammatory pathways (Furuhashi et al., 2014[32]). FABP4 generally had higher affinity and selectivity for long-chain fatty acids and palmitic acid, a saturated fatty acid, that has relatively high affinity for FABP4 under a specific condition such as obesity-induced oxidative stress (Furuhashi, 2019[31]). Palmitic acid can be toxic to cardiomyocytes producing cardiomyocyte cell death (Kong and Rabkin, 2002[59]).

FABP4 plasma levels predicted a higher risk for the development of heart failure as demonstrated in the Cardiovascular Health Study, a large epidemiologic study with a median follow-up period of 10.7 years (Djousse et al., 2013[24]). This association was attenuated but remained statistically significant upon adjustment for traditional HF risk factors including BMI and eGFR (Djousse et al., 2013[24]). As well, there was no evidence that this association was modified after consideration of ethnicity, age, sex, waist circumference, and diabetes status (Djousse et al., 2013[24]). The investigators concluded that the relationship was the same for HFpEF and HFrEF (Djousse et al., 2013[24]). The concept has developed that FABP4 is a predictor but not a causative factor of HF (Rodriguez-Calvo et al., 2017[94]). There is however, some experimental evidence suggesting that the metabolic effects of FABP4 should be able to modify cardiac function (Rodriguez-Calvo et al., 2017[94]). Interestingly, fatty acid-binding protein 4 which differentiated women with diastolic dysfunction from controls was also one of 10 biomarkers that differentiated women with pre-eclampsia from controls (Alma et al., 2017[2]). There are, however, not a lot of data on FABP4 in HFpEF and it should be an area of further research.

### sST2 

In the analysis of the KaRen study, Hedman et al., reported that ST2 was significantly elevated in **Phenogroup 2** that also had the worst outcome (Hedman et al., 2020[43]). ST2 is part of the interleukin-1 receptor family (IL-1R) which binds IL-33 using IL-1R3 as co-receptor (Boraschi and Tagliabue, 2013[9]; Pascual-Figal and Januzzi, 2015[82]). The soluble isoform (sST2) is one of several ST2 isoforms, which include both it and a transmembrane isoform (ST2L) generated by alternative splicing (Iwahana et al., 1999[49]. IL-33, an interleukin-1-like cytokine, signals via the IL-1 receptor-related protein ST2 and induces T-helper type 2-associated cytokines (Schmitz et al., 2005[106]). IL-1R4, the IL-33 binding chain, is known as T1 or ST2 (Boraschi and Tagliabue, 2013[9]). ST2 is up-regulated in cardiac myocytes by mechanical strain or myocardial injury (Weinberg et al., 2002[129]; Sanada et al., 2007[101]; Pascual-Figal and Januzzi, 2015[82]). IL-33/ST2 exerts beneficial effects on the myocardium by limiting cardiomyocyte cell death, hypertrophy and preventing cardiac fibrosis (Sanada et al., 2007[101]; Kakkar and Lee, 2008[51]; Seki et al., 2009[110]; Pascual-Figal and Januzzi, 2015[82]). The source of circulating ST2 in heart failure originates mainly in the heart but also in the lungs and the vascular endothelium (Pascual-Figal et al., 2018[83]). sST2 has been proposed to be a surrogate of pulmonary congestion in heart failure (Bayes-Genis et al., 2018[7]). We have reviewed the data on sST2 in HFpEF (Rabkin and Tang, 2021[91]) and found that sST2 correlates with the severity of LV diastolic dysfunction (Wang et al., 2013[127]; Zile et al., 2015[134]; Ruocco et al., 2019[97]). However, we reported (Rabkin and Tang, 2021[91]) there was no significant difference between HFpEF and HFrEF (Santhanakrishnan et al., 2012[105]; Sanders-van Wijk et al., 2015[102]; Sinning et al., 2017[114]; Tromp et al., 2017[121]; Ruocco et al., 2019[97]) suggesting that sST2 does not have a unique role in HFpEF but maybe operative across HF types.

### Renin

Renin was a biomarker for high-risk groups. Whether renin is a cause or an effect of HFpEF remains to be determined. There are data that suggest a causal link between circulating renin and the underlying factors leading to HFpEF. High renin levels can increase left ventricular mass (Koga et al., 1998[58]) either directly or through increases in aldosterone levels (Edelmann et al., 2012[27]). Renin has also been linked to myocardial fibrosis (Nguyen and Danser, 2008[78]). Circulating renin contributes to cardiac-specific synthesis of angiotensin peptides that in turn enhance cardiac fibrosis (Prescott et al., 2000[86]).

### TNF receptors

A number of TNF receptor types were elevated in the HFpEF subtype with the worst prognosis. There is a need for further investigation into their interrelationship and whether they might play a causal role in HFpEF. Viewed within the concept that HFpEF is related to a stiffer left ventricle produced by either cardiac hypertrophy or fibrosis, it is noteworthy that soluble tumor necrosis factor receptor (sTNFR)1 was significantly associated with LV mass, in a large epidemiologic study, even after multivariate analysis adjusting for demographic and medical risk factors (Takei et al., 2009[116]). In patients with hypertension and left ventricular hypertrophy, plasma sTNF-R1 was an independent predictor of left ventricular mass (Rosello-Lleti et al., 2009[95]). Development of cardiac fibrosis in response to angiotensin-II in mice consisted of two stages; an initial inflammatory response was followed by a fibrotic response; the latter was dependent in part on TNFR1 signaling (Duerrschmid et al., 2015[26]).

In the multicenter PROMIS-HFpEF study (Prevalence of Microvascular Dysfunction in Heart Failure With Preserved Ejection Fraction), 248 unique circulating proteins were quantified by a multiplex immunoassay (Sanders-van Wijk et al., 2020[102]). TNFR1 and GDF-15 as well as UPAR (urokinase plasminogen activator receptor), IGFBP7 (insulin-like growth factor binding protein 7) were the top individual proteins that mediated the relationship between comorbidity burden and echocardiographic parameters of HFpEF (Sanders-van Wijk et al., 2020[102]).

### Matrix metalloproteinases and their inhibitors

Matrix metalloproteinases have been linked to various cardiovascular diseases (Rabkin, 2014[88]; DeLeon-Pennell et al., 2017[22]) and appear to be relevant in HFpEF. Increased circulating matrix metalloproteinase-2 (MMP-2), the MMP tissue inhibitor-4 (TIMPS-4 and collagen III *N*-terminal propeptide [PIIINP]) along with decreased MMP-8 predict the presence of diastolic heart failure (Zile et al., 2011[135]). Indeed, a panel of these biomarkers, performed better than any single biomarker including NT-proBNP at identifying LVH or HFpEF (Zile et al., 2011[135]). Elevated level of the active form of MMP-9 is associated with diastolic dysfunction, and the level of elevation correlates with the severity of diastolic dysfunction in patients with CAD (Chu et al., 2011[17]).

### Integration of clinical characteristics of phenogroups and biomarkers with poor outcomes

Integrating the data on the clinical characteristics is important. Older **age** was significantly associated with a poorer outcome in most studies. **Sex** was a significant factor but in only 80 % of phenogroups showing a significant adverse outcome with females. 

***High BMI or obesity*** was reported in all but one study and of those it was considered to be a significant factor in 63 % (5 of 8) and was not significant in 38 % (3 of 8). The difference in the relevance of obesity is striking as some studies highlight it as a dominant characteristic of its phenotype with the worst outcome (Cohen et al., 2020[18]) while other studies did not have a high prevalence of obesity or high BMI in their phenotypes with the worst outcome (Kao et al., 2015[52]; Shah et al., 2015[112]; Nouraei and Rabkin, 2021[79]). The pathophysiologic explanations for the adverse effect of obesity center on the presence of a systemic inflammatory state in obesity as adipose tissue is infiltrated by macrophages that secrete pro-inflammatory cytokines (Taube et al., 2012[118]). In myocardium of HFpEF patients and ZSF1-HFpEF rats, E-selectin and intercellular adhesion molecule-1 expression levels were up-regulated; NADPH oxidase 2 expression was increased in endothelial cells and there was uncoupling of endothelial nitric oxide synthase. These data suggest that HFpEF is associated with coronary microvascular endothelial activation and oxidative stress leading to cardiomyocyte stiffness and cardiac hypertrophy (Franssen et al., 2016[30]). 

Interestingly, mice in which HFpEF was produced by *d*-aldosterone infusion, unilateral nephrectomy, and 1 % saline for 4 weeks, failed to regulate body temperature during cold temperature exposure despite a larger brown adipose tissue mass (Valero-Munoz et al., 2016[125]). These data suggest that HFpEF is associated with the expression and activation of the brown fat-specific genes in white adipocytes or beiging in white adipose tissue and with dysfunctional brown adipose tissue (Valero-Munoz et al., 2016[125]). Bariatric surgery is an effective method for weight reduction and reduction of epicardial fat (Rabkin and Campbell, 2015[89]) and it improves cardiac structure and left ventricular diastolic function (Kurnicka et al., 2018[61]).

***Hypertension*** was a significant factor in most studies (88 %, 7/8) in which it was reported. The development of HFpEF in hypertension is operative through several different pathways. First, hypertension-induced left ventricular hypertrophy represents a stiffer left ventricle because of the increased left ventricular mass. However, LVH is not always present in patients with HFpEF (Zile et al., 2011[136]) and different types of remodeling patterns (including eccentric LVH and concentric remodeling) can be found in patients with hypertension with or without HFpEF (Kasiakogias et al., 2021[54]). Thus, additional factors need to be implicated. One thesis is that hypertension along with the other factors such as obesity, DM, CKD and COPD (chronic obstructive pulmonary disease), induce a pro-inflammatory state resulting in coronary microvascular endothelial dysfunction, reduced bioavailability of nitric oxide, reduced cyclic guanosine monophosphate, and reduced protein kinase G (PKG) activity in the cardiomyocytes, that leads to increased LV stiffness (Paulus and Tschöpe, 2013[84]; Lee and Park, 2021[62]). This concept is supported by findings that patients with hypertension and HFpEF, but not patients with isolated hypertension, have significant increases in passive myocardial stiffness, collagen-dependent and titin-dependent stiffness in addition to changes in titin phosphorylation (Zile et al., 2015[134]). Titin (also known as connectin), anchors the Z-disc and extends to the M-line region of the sarcomere (Granzier and Labeit, 2004[36]). It functions as a molecular spring, maintaining the structural arrangement of thick and thin filaments, so that it plays a major role in passive muscle stiffness; an important determinant of diastolic function (Granzier and Labeit, 2004[36]).

Other factors are undoubtedly also operative. Aldehyde dehydrogenase 2 (ALDH2) rs671 polymorphism, a genetic risk factor for hypertension, in Asian populations is also associated with an increased risk of HFpEF (Xia et al., 2020[132]).

Establishing target blood pressures for antihypertensive drug therapy would be helpful to establish the role of hypertension in HFpEF. However to date no study has directly investigated the optimal blood pressure target in patients with hypertension and HFpEF (Kasiakogias et al., 2021[54]). Just as low blood pressure treatment can have adverse consequences for patients with hypertension (Rabkin et al., 2013[92]; Khan et al., 2018[57]), in older patients with hypertension and HFpEF, a systolic BP of less than 120 mmHg was associated with a higher risk of death (Faselis et al., 2021[29]).

***Atrial fibrillation*** was also a significant factor in the phenotypes with adverse outcomes as it was a significant factor in most studies (89 %; 8/9) in which it was reported. In patients hospitalized with HFpEF in the Japanese heart failure syndrome with preserved ejection fraction Nationwide Multicenter Registry, the incidence of adverse events was higher in the AF group without CAD than non-AF without CAD in multivariate analysis after consideration of other factors (Temma et al., 2020[119]). Older patients with HFpEF and atrial fibrillation, in one study had the highest risk one year mortality (HR 1.71) (Tromp et al., 2018[122]). The link between atrial fibrillation and HFpEF may represent one or both of two mechanisms which operate to produce increased stiffness of the left ventricle and atrial fibrillation specifically inflammation and/or cardiac fibrosis. Patients with HFpEF have increased serum levels of pro-inflammatory cytokines as discussed above (Table **3**(Tab. 3)). Inflammation can also activate fibrotic pathways leading to cardiac fibrosis with structural re-modeling of the atria (Hu et al., 2015[46]). Atrial fibrosis has been demonstrated by cardiac MRI in AF (Gal and Marrouche, 2017[33]). Cardiac fibroblasts can establish contact with cardiomyocytes developing low-resistance electrical junctions that can enhance phase 4 depolarization and promote ectopic impulse formation leading to re-entrant arrhythmias (Nattel, 2017[77]).

***Diabetes mellitus*** status was available in all studies and was a significant factor in the phenotypes with the worst outcome in 78 % of the studies. Clinical studies support the finding that the presence of diabetes mellitus worsens the prognosis of patients with HFpEF and also identify that patients with DM have more cardiac fibrosis as identified on MRI (Chirinos et al., 2019[15]; Lejeune et al., 2021[65]). Patients with HFpEF and DM have greater aortic stiffness as measured by carotid-femoral pulse wave velocity indicating a greater load on the left ventricle (Chirinos et al., 2019[13]). Patients with type 2 DM and HFpEF tend to have worse LV diastolic function (Wang et al., 2018[126]). Patients with type 2 DM and HFpEF show higher BMI and more kidney disease and anemia than those without type 2 DM (Arevalo-Lorido et al., 2021[5]). Although DM is often associated with co-morbidities, in HFpEF DM is a significant predictor of mortality and hospitalization for HF even after adjusting for factors such as age, BMI, NYHA class and renal function (Lejeune et al., 2021[65]). In the I-Preserve trial (Irbesartan in Heart Failure With Preserved Ejection Fraction), over a follow-up of 4.1 years, cardiovascular death or heart failure hospitalization occurred in 34 % of patients with diabetes mellitus versus 22 % of those without diabetes mellitus (adjusted hazard ratio, 1.75), and total mortality was greater in patients compared to those without diabetes mellitus (28 % versus 19 %; adjusted hazard ratio, 1.59) (Kristensen et al., 2017[60]). These adverse outcomes for HFpEF patients with DM are accentuated by the presence of microvascular complications (Sandesara et al., 2018[104]).

Several common pathological mechanisms in HFpEF and DM, including sodium retention and metabolic derangements link DM to HFpEF (McHugh et al., 2019[72]). In human diabetic hearts, titin hypophosphorylation at S4099 and hyperphosphorylation at S11878 suggest increased passive cardiomyocyte tension as well as altered activity of protein kinases (Hopf et al., 2018[45]).

In TOPCAT, the presence of DM was associated with higher levels of cardiac pro-fibrotic, and pro-inflammatory biomarkers. High-sensitivity C-reactive protein, pro-collagen type III amino-terminal peptide, tissue inhibitor of metalloproteinase 1 (TIMP-1), and galectin-3 levels were higher in persons with DM than those without diabetes. There was a significant increase in levels of high-sensitivity troponin T (hs-TnT), a marker of myocyte death, in DM patients. Elevated pro-collagen type III amino-terminal peptide and galectin-3 levels were associated with an increased risk of the primary outcome (cardiovascular mortality, aborted cardiac arrest, or HF hospitalization) in DM patients, but not in those without diabetes (De Marco et al., 2021[21]).

There are a number of potential mechanisms that may be operative in patients with DM leading to HFpEF. Coronary (micro)vascular dysfunction and lymphatic vessel alterations can play a role in HFpEF by reducing cardiac perfusion, and producing chronic low-grade inflammation, and myocardial edema, fibrosis, and cardiomyocyte stiffness (Cuijpers et al., 2020[20]). Coronary microvascular rarefaction may contribute to the left ventricular diastolic dysfunction and impaired cardiac reserve function characteristic of HfpEF (Mohammed et al., 2015[74]).

Metformin treatment was associated with a lower incidence of new-onset symptomatic HFpEF, LV diastolic dysfunction and hypertrophy in patients with type 2 DM and hypertension (Gu et al., 2020[38]).

***CAD ***was available in only 8 of 10 studies and was a significant factor in 5 (of 8) or 63 % of studies. In other literature, the impact of CAD in HFpEF prognosis is not clear. Several observational studies did not show a prognostic impact of CAD on HFpEF while other studies found a high prevalence of CAD in HFpEF compared with patients with LV hypertrophy without heart failure or controls (Ohara and Little, 2010[80]). One study found that patients with HFpEF and CAD had a greater deterioration in ejection fraction and increased mortality, independent of other predictors (hazard ratio: 1.71) compared with patients without CAD (Hwang et al., 2014[47]). In another study of patients followed for 10 years, patients with HFpEF and CAD were found to be at high risk of cardiovascular death, especially sudden death (Rusinaru et al., 2014[98]). Elevated circulating level of the active form of MMP-9 and TIMP-1 increased the ability to discriminate patients with HFpEF from controls (Chu et al., 2011[17]). This finding has raised the suggestion that abnormal extracellular matrix metabolism reflects the extent of myocardial ischemia (Chu et al., 2011[17]).

Some of the patients with HFpEF and symptoms of myocardial ischemia have small vessel disease rather than epicardial coronary stenosis. Cardiac MRI studies have concluded that HFpEF patients have a high prevalence of coronary microvascular dysfunction (CMD) as well as diffuse fibrosis (Loffler et al., 2019[67]). The microvascular disease may in part be due to increased vascular stiffness. In multivariable linear regression analysis, vascular stiffness assessed by pulse wave velocity was an independent predictor of diastolic function, as defined by left ventricular early diastolic circumferential strain rate (Samuel et al., 2021[100]). In symptomatic patients without overt CAD, impaired coronary flow reserve was independently associated with diastolic dysfunction and adverse events, especially HFpEF hospitalization (Taqueti and Di Carli, 2018[117]). The presence of both coronary microvascular and diastolic dysfunctions was associated with an increased risk of HFpEF events (Taqueti and Di Carli, 2018[117]).

***CKD ***status was available in all except one study and was a significant factor in all of those studies. Renal dysfunction in HFpEF is a consequence of the complex interplay between hemodynamic factors, systemic congestion, inflammation, endothelial dysfunction, and neurohormonal mechanisms. (Seetharam et al., 2020[108]). CHD maybe causally related to HFpEF and as well HFpEF may exacerbate CKD, worsening renal function in patients hospitalized with HFpEF (Sharma et al., 2015[113]). 

Patient with HFpEF and CKD have worse diastolic dysfunction as reflected by increased left atrial (LA) reservoir strain, LV longitudinal strain, and right ventricular free wall strain even after adjusting for potential confounders, including co-morbidities, EF, and volume status (Unger et al., 2016[123]). 

CKD is a predictor for the development of HFpEF in patients with subclinical diastolic dysfunction (Kaptein et al., 2020[53]). CKD in patients with HFpEF is characterized by echocardiographic and biomarker profiles indicative of more advanced heart disease (Mavrakanas et al., 2019[71]). Patients with CKD were at increased risk for HFpEF admission (Mavrakanas et al., 2019[71]). 

In patients with HFpEF, elevated cystatin C levels were associated with higher incidence of all-cause mortality and hospital readmissions, outperforming other estimates of renal function including eGFR, BUN and creatinine (Carrasco-Sanchez et al., 2011[11]).

Renal impairment causes metabolic and systemic derangements in circulating factors, causing an activated systemic inflammatory state and endothelial dysfunction, which may lead to cardiomyocyte stiffening, hypertrophy, and interstitial fibrosis via cross-talk between the endothelium and cardiomyocyte compartments (ter Maaten et al., 2016[120]).

Patients with CKD have a different metabolic profile consistent with increased inflammation and oxidative stress, impaired lipid metabolism, increased collagen synthesis, and down-regulated nitric oxide signaling (Hage et al., 2020[41]).

**Clinical symptoms (NYHA class) or the degree of BNP (NT-BNP) elevations** were a significant factor in all 7 of the studies in which it was analyzed. This likely reflects the degree of diastolic dysfunction or increase in left ventricular end diastolic or left atrial pressure in patients with HFpEF. 

**COPD** was available in only 8 of 10 studies and was a significant factor in 3 or 33 % of the studies. Although in one of the studies, COPD was in the high risk phenotype showed (Woolley et al., 2021[131]), the overall data do not support the contention that COPD is an indicator of an adverse outcome in HFpEF.

### Clinical factors to predict outcome in HfpEF MAGGIC score

**Figure 3:**
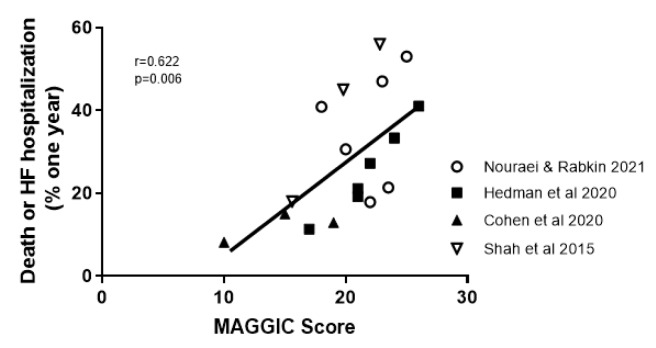
Correlation between the MAGGIC score in the papers that calculated it and the one year outcome

The MAGGIC score is based on individual data on 39,372 patients with HF, both reduced and preserved left-ventricular ejection fraction, from 30 cohort studies (Pocock et al., 2013[85]). It identified thirteen significant independent predictors of mortality which were (in order of predictive strength): age, lower EF, NYHA class, serum creatinine, diabetes, not prescribed beta-blocker, lower systolic BP, lower body mass, time since diagnosis, current smoker, chronic obstructive pulmonary disease, male gender, and not prescribed ACE-inhibitor or angiotensin-receptor blockers (Pocock et al., 2013[85]). In HFpEF, age was more predictive and systolic BP was less predictive of mortality than in persons with reduced EF (Pocock et al., 2013[85]). Four studies, examined herein, calculated the MAGGIC score on their participants. Each study showed a statistically significant difference across phenogroups. Combining all studies showed a significant relationship between MAGGIC score and outcome, using the primary outcome of each study and a one-year event rate (Figure 3(Fig. 3); References in Figure 3: Cohen et al., 2020[18]; Hedman et al., 2020[43]; Nouraei and Rabkin, 2021[79]; Shah et al., 2015[112]).

The phenogroup approach carries additional useful data. In TOPCAT, **Phenogroup 3** predicted outcome even after accounting for the MAGGIC score of that phenogroup (Cohen et al., 2020[18]).

### A new simplified formula to assess adverse outcome in HFpEF based on clinical factors

**Figure 4:**
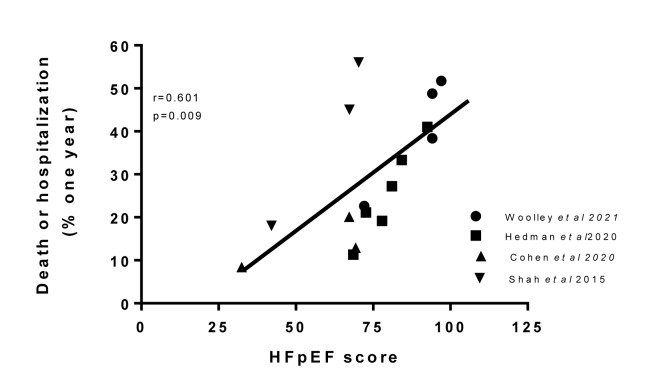
Correlation between the proposed HFpEF clinical score, in publications in which it could be calculated, and the one year outcome

The MAGGIC score, however, was derived mainly from patients with HFrEF and not HFpEF. It also considered medications which can readily be changed and may not be as relevant in HFpEF. Utilizing the data from all 10 studies with HFpEF, examined herein showed that there was a gradation in the contribution of each of ten clinical variables from those that were significant in all studies (e.g. age and CKD), to those marginally more common in those with poor outcome (diabetes mellitus) to conditions that usually were not significantly related to an adverse outcome in the majority of studies, specifically COPD. Assigning a value to each of the clinical factors that were present in the phenotypes with the poorest outcome of 20, specifically age (over 75 years), hypertension, atrial fibrillation, chronic kidney disease and worse symptoms severity (NYHA class III or greater); 15 to factors with an adverse outcome that were present in 65 % to 85 % of studies female sex, diabetes mellitus as well as age 70 to 75 years, and a value of 10 when the factor was present in less than 65 % and more than 50 % of studies, namely obesity and coronary artery disease. There was a significant (p=0.009) correlation between this new HFpEF prognostic score and one year clinical outcome (Figure 4(Fig. 4); References in Figure 4: Cohen et al., 2020[18]; Hedman et al., 2020[43]; Shah et al., 2015[112]; Woolley et al., 2021[131]). 

## Conclusions

The application of machine learning strategies to patients with HFpEF have consistently identified discrete groups of individuals. The number of groups or phenogroups varies from three to six. The clinical findings associated with the different phenotypes in > 85 % of studies were age, hypertension, atrial fibrillation, chronic kidney disease and worse symptoms severity; an adverse outcome in 65 % to 85 % of studies was associated with diabetes mellitus and female sex and in less than 65 % of studies were the clinical factors - body mass index or obesity, and coronary artery disease. The MAGGIC score was available in four studies. Focusing on the HFpEF phenotypes with the worst prognosis, found that the MAGGIC score correlated significantly with poor outcome. A new and more simplified score, based on clinical factors, was proposed and correlated significantly with adverse outcome in HFpEF. Three studies examined biomarkers in detail in their patient phenogroups. Several biomarkers were consistently elevated in phenogroups with adverse outcomes and suggest the underlying mechanism or pathophysiology specific for phenotypes with an adverse prognosis.

## Declaration

### Conflict of interest

There are no conflicts of interest in this work.

## Figures and Tables

**Table 1:**
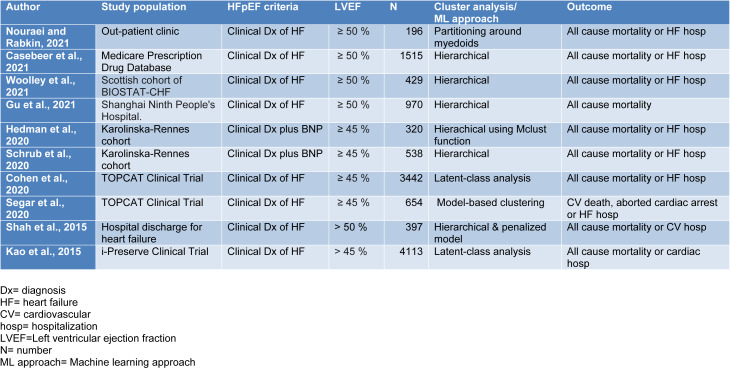
Nature of the studies

**Table 2:**
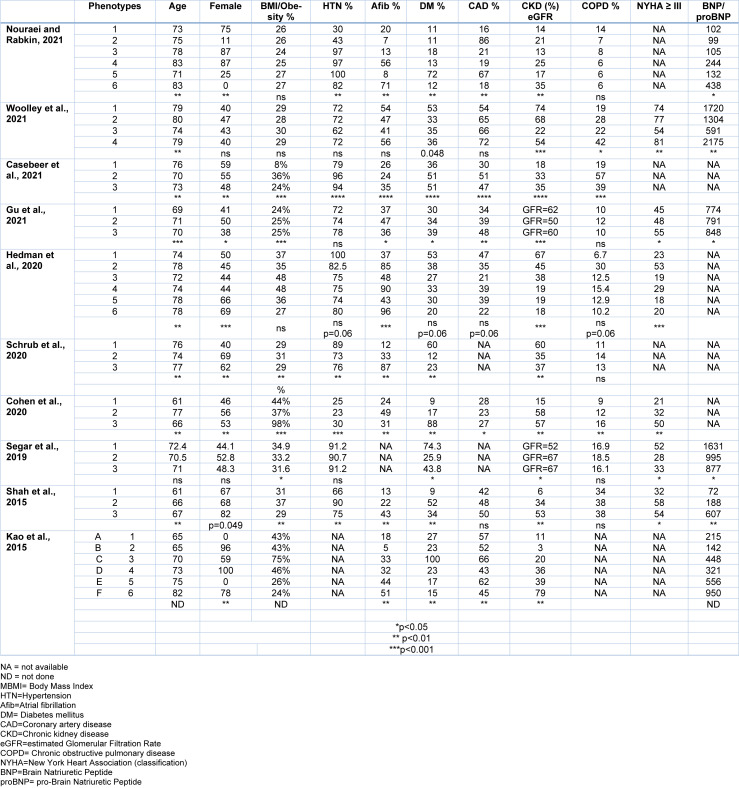
Clinical characteristics of each phenotype in the ten studies

**Table 3:**
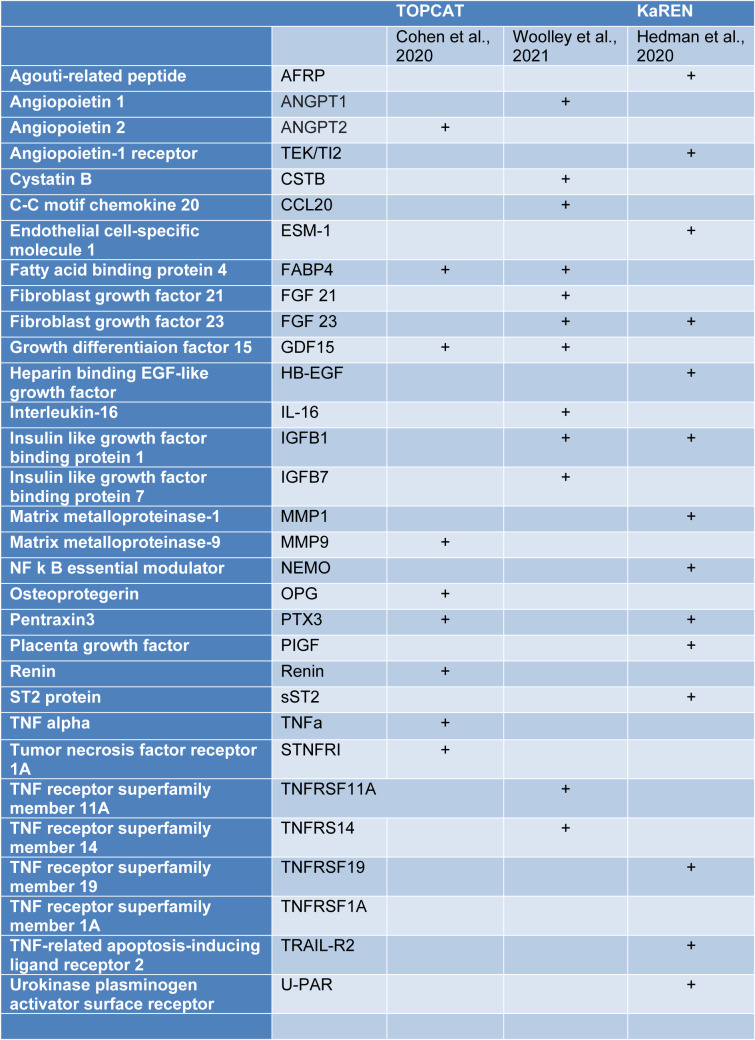
Some biomarkers associated with worst outcome in HFpEF

## References

[1] Albakri A (2018). Heart failure with preserved ejection fraction: A review of clinical status and meta-analysis of diagnosis by myocardial strain and effect of medication on mortality and hospitalization. Intern Med Care.

[2] Alma LJ, Bokslag A, Maas AHEM, Franx A, Paulus WJ, de Groot CJM (2017). Shared biomarkers between female diastolic heart failure and pre-eclampsia: a systematic review and meta-analysis. ESC Hear Fail.

[3] Almahmoud MF, Soliman EZ, Bertoni AG, Kestenbaum B, Katz R, Lima JAC (2018). Fibroblast growth factor-23 and heart failure with reduced versus preserved ejection fraction: MESA. J Am Heart Assoc.

[4] Angraal S, Mortazavi BJ, Gupta A, Khera R, Ahmad T, Desai NR (2020). Machine learning prediction of mortality and hospitalization in heart failure with preserved ejection fraction. JACC Heart Fail.

[5] Arevalo-Lorido JC, Carretero-Gomez J, Gomez-Huelgas R, Llacer P, Manzano L, Quesada Simon MA (2021). Comorbidities and their implications in patients with and without type 2 diabetes mellitus and heart failure with preserved ejection fraction. Findings from the rica registry. Int J Clin Pract.

[6] Baessler A, Strack C, Rousseva E, Wagner F, Bruxmeier J, Schmiedel M (2012). Growth-differentiation factor-15 improves reclassification for the diagnosis of heart failure with normal ejection fraction in morbid obesity. Eur J Heart Fail.

[7] Bayes-Genis A, Gonzalez A, Lupon J (2018). ST2 in heart failure. Circ Heart Fail.

[8] Bettencourt P, Ferreira-Coimbra J, Rodrigues P, Marques P, Moreira H, Pinto MJ (2018). Towards a multi-marker prognostic strategy in acute heart failure: a role for GDF-15. ESC Hear Fail.

[9] Boraschi D, Tagliabue A (2013). The interleukin-1 receptor family. Semin Immunol.

[10] Brouwers FP, De Boer RA, Van Der Harst P, Voors AA, Gansevoort RT, Bakker SJ (2013). Incidence and epidemiology of new onset heart failure with preserved vs. reduced ejection fraction in a community-based cohort: 11-year follow-up of PREVEND. Eur Heart J.

[11] Carrasco-Sanchez FJ, Galisteo-Almeda L, Paez-Rubio I, Martinez-Marcos FJ, Camacho-Vazquez C, Ruiz-Frutos C (2011). Prognostic value of cystatin C on admission in heart failure with preserved ejection fraction. J Card Fail.

[12] Casebeer A, Horter L, Hayden J, Simmons J, Evers T (2021). Phenotypic clustering of heart failure with preserved ejection fraction reveals different rates of hospitalization. J Cardiovasc Med (Hagerstown).

[13] Chirinos JA, Bhattacharya P, Kumar A, Proto E, Konda P, Segers P (2019). Impact of diabetes mellitus on ventricular structure, arterial stiffness, and pulsatile hemodynamics in heart failure with preserved ejection fraction. J Am Heart Assoc.

[14] Chirinos JA, Orlenko A, Zhao L, Basso MD, Cvijic ME, Li Z (2020). Multiple plasma biomarkers for risk stratification in patients with heart failure and preserved ejection fraction. J Am Coll Cardiol.

[15] Chirinos JA, Segers P, Hughes T, Townsend R (2019). Large-artery stiffness in health and disease: JACC state-of-the-art review. J Am Coll Cardiol.

[16] Chou R-H, Huang P-H, Hsu C-Y, Chang C-C, Leu H-B, Huang C-C (2016). Circulating fibroblast growth factor 21 is associated with diastolic dysfunction in heart failure patients with preserved ejection fraction. Sci Rep.

[17] Chu JW, Jones GT, Tarr GP, Phillips LV, Wilkins GT, van Rij AM (2011). Plasma active matrix metalloproteinase 9 associated to diastolic dysfunction in patients with coronary artery disease. Int J Cardiol.

[18] Cohen JB, Schrauben SJ, Zhao L, Basso MD, Cvijic ME, Li Z (2020). Clinical phenogroups in heart failure with preserved ejection fraction: detailed phenotypes, prognosis, and response to spironolactone. JACC Heart Fail.

[19] Cotter G, Voors AA, Prescott MF, Felker GM, Filippatos G, Greenberg BH (2015). Growth differentiation factor 15 (GDF-15) in patients admitted for acute heart failure: results from the RELAX-AHF study. Eur J Heart Fail.

[20] Cuijpers I, Simmonds SJ, van Bilsen M, Czarnowska E, Gonzalez Miqueo A, Heymans S (2020). Microvascular and lymphatic dysfunction in HFpEF and its associated comorbidities. Basic Res Cardiol.

[21] De Marco C, Claggett BL, de Denus S, Zile MR, Huynh T, Desai AS (2021). Impact of diabetes on serum biomarkers in heart failure with preserved ejection fraction: insights from the TOPCAT trial. ESC Hear Fail.

[22] DeLeon-Pennell KY, Meschiari CA, Jung M, Lindsey ML (2017). Matrix metalloproteinases in myocardial infarction and heart failure. Prog Mol Biol Transl Sci.

[23] Dinh W, Futh R, Lankisch M, Hess G, Zdunek D, Scheffold T (2011). Growth-differentiation factor-15: a novel biomarker in patients with diastolic dysfunction?. Arq Bras Cardiol.

[24] Djousse L, Bartz TM, Ix JH, Kochar J, Kizer JR, Gottdiener JS (2013). Fatty acid-binding protein 4 and incident heart failure: the Cardiovascular Health Study. Eur J Heart Fail.

[25] Donal E, Lund LH, Linde C, Edner M, Lafitte S, Persson H (2009). Rationale and design of the Karolinska-Rennes (KaRen) prospective study of dyssynchrony in heart failure with preserved ejection fraction. Eur J Heart Fail.

[26] Duerrschmid C, Trial J, Wang Y, Entman ML, Haudek SB (2015). Tumor necrosis factor: a mechanistic link between angiotensin-II-induced cardiac inflammation and fibrosis. Circ Heart Fail.

[27] Edelmann F, Tomaschitz A, Wachter R, Gelbrich G, Knoke M, Dungen H-D (2012). Serum aldosterone and its relationship to left ventricular structure and geometry in patients with preserved left ventricular ejection fraction. Eur Heart J.

[28] Fairlie WD, Zhang HP, Wu WM, Pankhurst SL, Bauskin AR, Russell PK (2001). The propeptide of the transforming growth factor-beta superfamily member, macrophage inhibitory cytokine-1 (MIC-1), is a multifunctional domain that can facilitate protein folding and secretion. J Biol Chem.

[29] Faselis C, Lam PH, Zile MR, Bhyan P, Tsimploulis A, Arundel C (2021). Systolic blood pressure and outcomes in older patients with HFpEF and hypertension. Am J Med.

[30] Franssen C, Chen S, Unger A, Korkmaz HI, De Keulenaer GW, Tschope C (2016). Myocardial microvascular inflammatory endothelial activation in heart failure with preserved ejection fraction. JACC Heart Fail.

[31] Furuhashi M (2019). Fatty acid-binding protein 4 in cardiovascular and metabolic diseases. J Atheroscler Thromb.

[32] Furuhashi M, Saitoh S, Shimamoto K, Miura T (2014). Fatty acid-binding protein 4 (FABP4): pathophysiological insights and potent clinical biomarker of metabolic and cardiovascular diseases. Clin Med Insights Cardiol.

[33] Gal P, Marrouche NF (2017). Magnetic resonance imaging of atrial fibrosis: redefining atrial fibrillation to a syndrome. Eur Heart J.

[34] Ghuman J, Cai X, Patel RB, Khan SS, Hecktman J, Redfield MM (2021). Fibroblast growth factor 23 and exercise capacity in heart failure with preserved ejection fraction. J Card Fail.

[35] Giorgio Q, Ramy A, Michael H, Rima A (2021). Machine learning and the future of cardiovascular care. J Am Coll Cardiol.

[36] Granzier HL, Labeit S (2004). The giant protein titin: a major player in myocardial mechanics, signaling, and disease. Circ Res.

[37] Gu J, Pan J-A, Lin H, Zhang J-F, Wang C-Q (2021). Characteristics, prognosis and treatment response in distinct phenogroups of heart failure with preserved ejection fraction. Int J Cardiol.

[38] Gu J, Yin Z-F, Zhang J-F, Wang C-Q (2020). Association between long-term prescription of metformin and the progression of heart failure with preserved ejection fraction in patients with type 2 diabetes mellitus and hypertension. Int J Cardiol.

[39] Guo H, Zhao X, Li H, Liu K, Jiang H, Zeng X (2021). GDF15 promotes cardiac fibrosis and proliferation of cardiac fibroblasts via the MAPK/ERK1/2 pathway after irradiation in rats. Radiat Res.

[40] Gustafsson F, Torp-Pedersen C, Brendorp B, Seibæk M, Burchardt H, Køber L (2003). Long-term survival in patients hospitalized with congestive heart failure: Relation to preserved and reduced left ventricular systolic function. Eur Heart J.

[41] Hage C, Linde C, Lofgren L, Nilsson R, Davidsson P, Kumar C (2020). Metabolomic profile in HFpEF vs HFrEF patients. J Card Fail.

[42] Hastie T, Tibshirani R, Friedman J (2009). The elements of statistical learning.

[43] Hedman ÅK, Hage C, Sharma A, Brosnan MJ, Buckbinder L, Gan L-M (2020). Identification of novel pheno-groups in heart failure with preserved ejection fraction using machine learning. Heart.

[44] Heger J, Schiegnitz E, von Waldthausen D, Anwar MM, Piper HM, Euler G (2010). Growth differentiation factor 15 acts anti-apoptotic and pro-hypertrophic in adult cardiomyocytes. J Cell Physiol.

[45] Hopf A-E, Andresen C, Kotter S, Isic M, Ulrich K, Sahin S (2018). Diabetes-induced cardiomyocyte passive stiffening is caused by impaired insulin-dependent titin modification and can be modulated by neuregulin-1. Circ Res.

[46] Hu Y-F, Chen Y-J, Lin Y-J, Chen S-A (2015). Inflammation and the pathogenesis of atrial fibrillation. Nat Rev Cardiol.

[47] Huang Z (1998). Extensions to the k-means algorithm for clustering large data sets with categorical values. Data Min Knowl Discov.

[48] Hwang S, Melenovsky V, Borlaug B (2014). Implications of coronary artery disease in heart failure with preserved ejection fraction. J Am Coll Cardiol.

[49] Iwahana H, Yanagisawa K, Ito-Kosaka A, Kuroiwa K, Tago K, Komatsu N (1999). Different promoter usage and multiple transcription initiation sites of the interleukin-1 receptor-related human ST2 gene in UT-7 and TM12 cells. Eur J Biochem.

[50] Jin Y-J, Lee J-H, Kim Y-M, Oh GT, Lee H (2012). Macrophage inhibitory cytokine-1 stimulates proliferation of human umbilical vein endothelial cells by up-regulating cyclins D1 and E through the PI3K/Akt-, ERK-, and JNK-dependent AP-1 and E2F activation signaling pathways. Cell Signal.

[51] Kakkar R, Lee RT (2008). The IL-33/ST2 pathway: therapeutic target and novel biomarker. Nat Rev Drug Discov.

[52] Kao DP, Lewsey JD, Anand IS, Massie BM, Zile MR, Carson PE (2015). Characterization of subgroups of heart failure patients with preserved ejection fraction with possible implications for prognosis and treatment response. Eur J Heart Fail.

[53] Kaptein YE, Karagodin I, Zuo H, Lu Y, Zhang J, Kaptein JS (2020). Identifying phenogroups in patients with subclinical diastolic dysfunction using unsupervised statistical learning. BMC Cardiovasc Disord.

[54] Kasiakogias A, Rosei EA, Camafort M, Ehret G, Faconti L, Ferreira JP (2021). Hypertension and heart failure with preserved ejection fraction: position paper by the European Society of Hypertension. J Hypertens.

[55] Kaufman, Rousseeuw P (1990). Finding groups in data. an introduction to cluster analysis.

[56] Kempf T, Eden M, Strelau J, Naguib M, Willenbockel C, Tongers J (2006). The transforming growth factor-beta superfamily member growth-differentiation factor-15 protects the heart from ischemia/reperfusion injury. Circ Res.

[57] Khan NA, Rabkin SW, Zhao Y, McAlister FA, Park JE, Guan M (2018). Effect of lowering diastolic pressure in patients with and without cardiovascular disease: analysis of the SPRINT (Systolic Blood Pressure Intervention Trial). Hypertension.

[58] Koga M, Sasaguri M, Miura S, Tashiro E, Kinoshita A, Ideishi M (1998). Plasma renin activity could be a useful predictor of left ventricular hypertrophy in essential hypertensives. J Hum Hypertens.

[59] Kong JY, Rabkin SW (2002). Palmitate-induced cardiac apoptosis is mediated through CPT-1 but not influenced by glucose and insulin. Am J Physiol Heart Circ Physiol.

[60] Kristensen SL, Mogensen UM, Jhund PS, Petrie MC, Preiss D, Win S (2017). Clinical and echocardiographic characteristics and cardiovascular outcomes according to diabetes status in patients with heart failure and preserved ejection fraction: a report from the I-preserve trial (Irbesartan in heart failure with preserved ejection. Circulation.

[61] Kurnicka K, Domienik-Karlowicz J, Lichodziejewska B, Bielecki M, Kozlowska M, Goliszek S (2018). Improvement of left ventricular diastolic function and left heart morphology in young women with morbid obesity six months after bariatric surgery. Cardiol J.

[62] Lee CJ, Park S (2021). Hypertension and heart failure with preserved ejection fraction. Heart Fail Clin.

[63] Leifheit-Nestler M, Haffner D (2018). Paracrine effects of FGF23 on the heart. Front Endocrinol (Lausanne).

[64] Leifheit-Nestler M, Wagner MA, Richter B, Piepert C, Eitner F, Bockmann I (2021). Cardiac fibroblast growth factor 23 excess does not induce left ventricular hypertrophy in healthy mice. Front Cell Dev Biol.

[65] Lejeune S, Roy C, Slimani A, Pasquet A, Vancraeynest D, Vanoverschelde J-L (2021). Diabetic phenotype and prognosis of patients with heart failure and preserved ejection fraction in a real life cohort. Cardiovasc Diabetol.

[66] Lin J-F, Wu S, Hsu S-Y, Yeh K-H, Chou H-H, Cheng S-T (2014). Growth-differentiation factor-15 and major cardiac events. Am J Med Sci.

[67] Loffler AI, Pan JA, Balfour PCJ, Shaw PW, Yang Y, Nasir M (2019). Frequency of coronary microvascular dysfunction and diffuse myocardial fibrosis (measured by cardiovascular magnetic resonance) in patients with heart failure and preserved left ventricular ejection fraction. Am J Cardiol.

[68] Lok S, Winkens, B, Goldschmeding R, van Geffen A, Nous F, van Kuik J, van der Weide P (2012). Circulating growth differentiation factor‐15 correlates with myocardial fibrosis in patients with non‐ischaemic dilated cardiomyopathy and decreases rapidly after left ventricular assist device support. Eur J Hear Fail.

[69] Magana-Serrano JA, Almahmeed W, Gomez E, Al-Shamiri M, Adgar D, Sosner P (2011). Prevalence of heart failure with preserved ejection fraction in Latin American, middle eastern, and North African regions in the i PREFER study (identification of patients with heart failure and PREserved systolic function: An epidemiological regional stu. Am J Cardiol.

[70] Martin A, David V, Quarles LD (2012). Regulation and function of the FGF23/klotho endocrine pathways. Physiol Rev.

[71] Mavrakanas TA, Khattak A, Wang W, Singh K, Charytan DM (2019). Association of chronic kidney disease with preserved ejection fraction heart failure is independent of baseline cardiac function. Kidney Blood Press Res.

[72] McHugh K, DeVore AD, Wu J, Matsouaka RA, Fonarow GC, Heidenreich PA (2019). Heart failure with preserved ejection fraction and diabetes: JACC state-of-the-art review. J Am Coll Cardiol.

[73] Mishra S, Kass DA (2021). Cellular and molecular pathobiology of heart failure with preserved ejection fraction. Nat Rev Cardiol.

[74] Mohammed SF, Hussain S, Mirzoyev SA, Edwards WD, Maleszewski JJ, Redfield MM (2015). Coronary microvascular rarefaction and myocardial fibrosis in heart failure with preserved ejection fraction. Circulation.

[75] Moher D, Liberati A, Tetzlaff J, Altman DG, PRISMA Group (2009). Preferred reporting items for systematic reviews and meta-analyses: The PRISMA statement. PLoS Med.

[76] Mushtaq H, Khawaja SG, Akram MU, Yasin A, Muzammal M, Khalid S (2018). A parallel architecture for the Partitioning Around Medoids (PAM) algorithm for scalable multi-core processor implementation with applications in healthcare. Sensors (Basel).

[77] Nattel S (2017). Molecular and cellular mechanisms of atrial fibrosis in atrial fibrillation. JACC Clin Electrophysiol.

[78] Nguyen G, Danser AHJ (2008). Prorenin and (pro)renin receptor: a review of available data from in vitro studies and experimental models in rodents. Exp Physiol.

[79] Nouraei H, Rabkin SW (2021). A new approach to the clinical subclassification of heart failure with preserved ejection fraction. Int J Cardiol.

[80] Ohara T, Little WC (2010). Evolving focus on diastolic dysfunction in patients with coronary artery disease. Curr Opin Cardiol.

[81] Owan TE, Hodge DO, Herges RM, Jacobsen SJ, Roger VL, Redfield MM (2006). Trends in prevalence and outcome of heart failure with preserved ejection fraction. N Engl J Med.

[82] Pascual-Figal DA, Januzzi JL (2015). The biology of ST2: the International ST2 Consensus Panel. Am J Cardiol.

[83] Pascual-Figal DA, Perez-Martinez MT, Asensio-Lopez MC, Sanchez-Mas J, Garcia-Garcia ME, Martinez CM (2018). Pulmonary production of soluble ST2 in heart failure. Circ Heart Fail.

[84] Paulus WJ, Tschöpe C (2013). A novel paradigm for heart failure with preserved ejection fraction: Comorbidities drive myocardial dysfunction and remodeling through coronary microvascular endothelial inflammation. J Am Coll Cardiol.

[85] Pocock SJ, Ariti CA, McMurry JJ, Maggioni A, Køber L, Squire IB (2013). Predicting survival in heart failure: a risk score based on 39 372 patients from 30 studies. Eur Heart J.

[86] Prescott G, Silversides DW, Chiu SM, Reudelhuber TL (2000). Contribution of circulating renin to local synthesis of angiotensin peptides in the heart. Physiol Genomics.

[87] Przewlocka-Kosmala M, Marwick TH, Dabrowski A, Kosmala W (2019). Contribution of cardiovascular reserve to prognostic categories of heart failure with preserved ejection fraction: a classification based on machine learning. J Am Soc Echocardiogr.

[88] Rabkin SW (2014). Differential expression of MMP-2, MMP-9 and TIMP proteins in thoracic aortic aneurysm–comparison with and without bicuspid aortic valve: A meta-analysis. Vasa.

[89] Rabkin SW, Campbell H (2015). Comparison of reducing epicardial fat by exercise, diet or bariatric surgery weight loss strategies: A systematic review and meta-analysis. Obes Rev.

[90] Rabkin SW, Sackett DL, Colman RW, Hirsh J, Marder VJ, Salzman ED (1982). Epidemiology of arterial thromboembolism. Hemostasis and thrombosis.

[91] Rabkin SW, Tang JKK (2021). The utility of growth differentiation factor-15, galectin-3, and sST2 as biomarkers for the diagnosis of heart failure with preserved ejection fraction and compared to heart failure with reduced ejection fraction: a systematic review. Heart Fail Rev.

[92] Rabkin SW, Waheed A, Poulter RS, Wood D (2013). Myocardial perfusion pressure in patients with hypertension and coronary artery disease: Implications for DBP targets in hypertension management. J Hypertens.

[93] Reddy YNV, Borlaug BA (2016). Heart failure with preserved ejection fraction. Curr Probl Cardiol.

[94] Rodriguez-Calvo R, Girona J, Alegret JM, Bosquet A, Ibarretxe D, Masana L (2017). Role of the fatty acid-binding protein 4 in heart failure and cardiovascular disease. J Endocrinol.

[95] Rosello-Lleti E, Rivera M, Martinez-Dolz L, Gonzalez Juanatey JR, Cortes R, Jordan A (2009). Inflammatory activation and left ventricular mass in essential hypertension. Am J Hypertens.

[96] Roy C, Lejeune S, Slimani A, de Meester C, Ahn As SA, Rousseau MF (2020). Fibroblast growth factor 23: a biomarker of fibrosis and prognosis in heart failure with preserved ejection fraction. ESC Hear Fail.

[97] Ruocco G, Evangelista I, Franci B, Lucani B, Martini S, Nuti R (2019). Combination of ST2 and B-type natriuretic peptide in diabetic patients with acute heart failure: relation with ventricular stiffness and outcome. J Cardiovasc Med.

[98] Rusinaru D, Houpe D, Szymanski C, Lévy F, Maréchaux S, Tribouilloy C (2014). Coronary artery disease and 10‐year outcome after hospital admission for heart failure with preserved and with reduced ejection fraction. Eur J Heart Fail.

[99] Sabbah MS, Fayyaz AU, de Denus S, Felker GM, Borlaug BA, Dasari S (2020). Obese-inflammatory phenotypes in heart failure with preserved ejection fraction. Circ Heart Fail.

[100] Samuel TJ, Wei J, Sharif B, Tamarappoo BK, Pattisapu V, Maughan J (2021). Diastolic dysfunction in women with ischemia and no obstructive coronary artery disease: Mechanistic insight from magnetic resonance imaging. Int J Cardiol.

[101] Sanada S, Hakuno D, Higgins LJ, Schreiter ER, McKenzie ANJ, Lee RT (2007). IL-33 and ST2 comprise a critical biomechanically induced and cardioprotective signaling system. J Clin Invest.

[102] Sanders-van Wijk S, Tromp J, Beussink-Nelson L, Hage C, Svedlund S, Saraste A (2020). Proteomic evaluation of the comorbidity-inflammation paradigm in heart failure with preserved ejection fraction: results from the PROMIS-HFpEF study. Circulation.

[103] Sanders-van Wijk S, van Empel V, Davarzani N, Maeder MT, Handschin R, Pfisterer ME (2015). Circulating biomarkers of distinct pathophysiological pathways in heart failure with preserved vs. reduced left ventricular ejection fraction. Eur J Heart Fail.

[104] Sandesara PB, O’Neal WT, Kelli HM, Samman-Tahhan A, Hammadah M, Quyyumi AA (2018). The prognostic significance of diabetes and microvascular complications in patients with heart failure with preserved ejection fraction. Diabetes Care.

[105] Santhanakrishnan R, Chong JPC, Ng TP, Ling LH, Sim D, Leong KTG (2012). Growth differentiation factor 15, ST2, high-sensitivity troponin T, and N-terminal pro brain natriuretic peptide in heart failure with preserved vs. reduced ejection fraction. Eur J Heart Fail.

[106] Schmitz J, Owyang A, Oldham E, Song Y, Murphy E, McClanahan TK (2005). IL-33, an interleukin-1-like cytokine that signals via the IL-1 receptor-related protein ST2 and induces T helper type 2-associated cytokines. Immunity.

[107] Schrub F, Oger E, Bidaut A, Hage C, Charton M, Daubert JC (2020). Heart failure with preserved ejection fraction: A clustering approach to a heterogenous syndrome. Arch Cardiovasc Dis.

[108] Seetharam K, Sengupta PP, Bianco CM (2020). Cardiac mechanics in heart failure with preserved ejection fraction. Echocardiography.

[109] Segar MW, Patel KV, Ayers C, Basit M, Tang WHW, Willett D (2020). Phenomapping of patients with heart failure with preserved ejection fraction using machine learning‐based unsupervised cluster analysis. Eur J Heart Fail.

[110] Seki K, Sanada S, Kudinova AY, Steinhauser ML, Handa V, Gannon J (2009). Interleukin-33 prevents apoptosis and improves survival after experimental myocardial infarction through ST2 signaling. Circ Heart Fail.

[111] Shah S (2017). Precision medicine for heart failure with preserved ejection fraction: an overview. J Cardiovasc Transl Res.

[112] Shah SJ, Katz DH, Selvaraj S, Burke MA, Yancy CW, Gheorghiade M (2015). Phenomapping for novel classification of heart failure with preserved ejection fraction. Circulation.

[113] Sharma K, Hill T, Grams M, Daya NR, Hays AG, Fine D (2015). Outcomes and worsening renal function in patients hospitalized with heart failure with preserved ejection fraction. Am J Cardiol.

[114] Sinning C, Kempf T, Schwarzl M, Lanfermann S, Ojeda F, Schnabel RB (2017). Biomarkers for characterization of heart failure - Distinction of heart failure with preserved and reduced ejection fraction. Int J Cardiol.

[115] Stahrenberg R, Edelmann F, Mende M, Kockskamper A, Dungen H-D, Luers C (2010). The novel biomarker growth differentiation factor 15 in heart failure with normal ejection fraction. Eur J Heart Fail.

[116] Takei Y, Di Tullio MR, Homma S, Boden-Albala B, Rundek T, Sacco RL (2009). Soluble tumor necrosis factor receptor 1 level is associated with left ventricular hypertrophy: the northern Manhattan study. Am J Hypertens.

[117] Taqueti VR, Di Carli MF (2018). Coronary microvascular disease pathogenic mechanisms and therapeutic options: JACC state-of-the-art review. J Am Coll Cardiol.

[118] Taube A, Schlich R, Sell H, Eckardt K, Eckel J (2012). Inflammation and metabolic dysfunction: links to cardiovascular diseases. Am J Physiol Heart Circ Physiol.

[119] Temma T, Nagai T, Watanabe M, Kamada R, Takahashi Y, Hagiwara H (2020). Differential prognostic impact of atrial fibrillation in hospitalized heart failure patients with preserved ejection fraction according to coronary artery disease status - report from the Japanese Nationwide Multicenter Registry. Circ J.

[120] ter Maaten JM, Damman K, Verhaar MC, Paulus WJ, Duncker DJ, Cheng C (2016). Connecting heart failure with preserved ejection fraction and renal dysfunction: the role of endothelial dysfunction and inflammation. Eur J Heart Fail.

[121] Tromp J, Khan MAF, Klip IjT, Meyer S, de Boer RA, Jaarsma T (2017). Biomarker profiles in heart failure patients with preserved and reduced ejection fraction. J Am Heart Assoc.

[122] Tromp J, Tay WT, Ouwerkerk W, Teng T-HK, Yap J, MacDonald MR (2018). Multimorbidity in patients with heart failure from 11 Asian regions: A prospective cohort study using the ASIAN-HF registry. PLoS Med.

[123] Unger ED, Dubin RF, Deo R, Daruwalla V, Friedman JL, Medina C (2016). Association of chronic kidney disease with abnormal cardiac mechanics and adverse outcomes in patients with heart failure and preserved ejection fraction. Eur J Heart Fail.

[124] Unsicker K, Spittau B, Krieglstein K (2013). The multiple facets of the TGF-beta family cytokine growth/ differentiation factor-15/macrophage inhibitory cytokine-1. Cytokine Growth Factor Rev.

[125] Valero-Munoz M, Li S, Wilson RM, Hulsmans M, Aprahamian T, Fuster JJ (2016). Heart failure with preserved ejection fraction induces beiging in adipose tissue. Circ Heart Fail.

[126] Wang Y, Yang H, Nolan M, Pathan F, Negishi K, Marwick TH (2018). Variations in subclinical left ventricular dysfunction, functional capacity, and clinical outcomes in different heart failure aetiologies. ESC Hear Fail.

[127] Wang Y-C, Yu C-C, Chiu F-C, Tsai C-T, Lai L-P, Hwang J-J (2013). Soluble ST2 as a biomarker for detecting stable heart failure with a normal ejection fraction in hypertensive patients. J Card Fail.

[128] Warbrick I, Rabkin SW (2019). Hypoxia-inducible factor 1-alpha (HIF-1α) as a factor mediating the relationship between obesity and heart failure with preserved ejection fraction. Obes Rev.

[129] Weinberg EO, Shimpo M, De Keulenaer GW, MacGillivray C, Tominaga S, Solomon SD (2002). Expression and regulation of ST2, an interleukin-1 receptor family member, in cardiomyocytes and myocardial infarction. Circulation.

[130] Wesseling M, de Poel JHC, de Jager SCA (2020). Growth differentiation factor 15 in adverse cardiac remodelling: from biomarker to causal player. ESC Hear Fail.

[131] Woolley RJ, Ceelen D, Ouwerkerk W, Tromp J, Figarska SM, Anker SD (2021). Machine learning based on biomarker profiles identifies distinct subgroups of heart failure with preserved ejection fraction. Eur J Heart Fail.

[132] Xia C-L, Chu P, Liu Y-X, Qu X-L, Gao X-F, Wang Z-M (2020). ALDH2 rs671 polymorphism and the risk of heart failure with preserved ejection fraction (HFpEF) in patients with cardiovascular diseases. J Hum Hypertens.

[133] Zhang Y, Moszczynski LA, Liu Q, Jiang J, Zhao D, Quan D (2017). Over-expression of growth differentiation factor 15 (GDF15) preventing cold ischemia reperfusion (I/R) injury in heart transplantation through Foxo3a signaling. Oncotarget.

[134] Zile MR, Baicu CF, Ikonomidis JS, Stroud RE, Nietert PJ, Bradshaw AD (2015). Myocardial stiffness in patients with heart failure and a preserved ejection fraction: contributions of collagen and titin. Circulation.

[135] Zile MR, Desantis SM, Baicu CF, Stroud RE, Thompson SB, McClure CD (2011). Plasma biomarkers that reflect determinants of matrix composition identify the presence of left ventricular hypertrophy and diastolic heart failure. Circ Heart Fail.

[136] Zile MR, Gottdiener JS, Hetzel SJ, McMurray JJ, Komajda M, McKelvie R (2011). Prevalence and significance of alterations in cardiac structure and function in patients with heart failure and a preserved ejection fraction. Circulation.

